# Generation of novel human anti-OX-40 mAbs endowed with different biological properties as tools for cancer therapy

**DOI:** 10.3389/fimmu.2025.1644391

**Published:** 2025-09-17

**Authors:** Rosa Rapuano Lembo, Margherita Passariello, Lorenzo Manna, Guendalina Froechlich, Martina Belardo, Alfredo Nicosia, Emanuele Sasso, Claudia De Lorenzo

**Affiliations:** ^1^ CEINGE-Biotecnologie avanzate Franco Salvatore S.C.a.R.L, Naples, Italy; ^2^ European School of Molecular Medicine, University of Milan, Milan, Italy; ^3^ Department of Molecular Medicine and Medical Biotechnologies, University of Naples “Federico II”, Naples, Italy

**Keywords:** antibodies, immunotherapy, immune checkpoints, OX-40, NK cells

## Abstract

**Introduction:**

The second generation of Antibody-based Immunotherapy includes monoclonal antibodies against Immune Checkpoints (ICs), to modulate specific T cell responses against cancer or viruses. We recently generated a large repertoire of fully human antibodies targeting ten different ICs through a novel selection strategy based on the combination of phage libraries on human lymphocytes and next generation sequencing (NGS). Here we generated and tested four novel fully human IgG1 mAbs specific for OX-40, an immunostimulatory receptor expressed on immune cells, which has been shown to be a promising target for immune-based therapeutic strategies.

**Methods and results:**

By ELISA and Biolayer Interferometry we demonstrated that they all specifically bind with high affinity to OX-40 and they recognize distinct epitopes. Three of them interfere with the binding of OX-40 and its ligand, thus suggesting that they compete with it for the receptor binding. T cell activation assays confirmed the agonistic properties of these 3 antibodies which are able to mimic the ligand by activating the pathway downstream the receptor. This activation results into an effective proliferation of hPBMCs and secretion of proinflammatory cytokines. Co-culture assays of hPBMCs with tumor cells confirm their ability to induce the activation of immune cells against cancer cells. The fourth antibody, even though non-agonistic, was able to induce the activation of lymphocytes by a different mechanism of action, based on NK-mediated Treg killing in co-culture assays.

**Discussion and conclusions:**

Combinations of these anti-OX40 mAbs targeting different epitopes lead to stronger activation of immune cells. Moreover, epitope binning analyses show that they recognize distinct epitopes not overlapping with that of the clinically validated Rocatinlimab, thus they could become potential new therapeutic tools. Taking advantage of the different behaviour of the novel mAbs, we also exploited them to clarify the unclear role of OX-40 on NK cells. We show here for the first time that NK cells express higher levels of a medium glycosylated OX-40 form than T cells, which is preferentially recognized by the novel mAbs but not by OX-40L, which instead binds to a highly glycosylated OX-40 variant absent on non-immune cells. Thus, glycosylation pattern could affect the recognition and biological effects of OX-40 binders and should be considered for the design of novel drugs.

## 1 Introduction

Cancer immunotherapy is a successful approach that exploits the ability of the immune system to detect and eradicate cancer cells (1). Negative and positive immune checkpoints (ICs) are physiologically engaged to regulate immune system homeostasis including cell proliferation, cytokines secretion, T cell activation and self-tolerance (2, 3). The blockade of negative immune checkpoints (ICs) by monoclonal antibodies (mAbs) has proven to be one of the most successful immunotherapeutic approaches. Several monoclonal antibodies blocking immune checkpoints such as Programmed cell Death-1 (PD-1), Programmed Death-Ligand 1 (PD-L1), Cytotoxic-T-Lymphocyte Antigen-4 (CTLA-4) have already been approved for the treatment of different types of cancer. Despite remarkable results, many patients develop primary or acquired resistance underlining the need for development of new therapeutic strategies. To overcome these hurdles, different arms of immune regulation can be simultaneously targeted in combination regimens involving both inhibitory and co-stimulatory immune checkpoints (2). OX-40, also known as CD134, is a receptor mainly expressed on the surface of activated T cells and endowed with co-stimulatory properties (4). OX40 intracellular signalling is activated upon it is engaged by its natural ligand (OX-40-OX-40-L), typically expressed by antigen-presenting cells (APCs) (5). OX-40/OX-40-L interaction promotes T cells proliferation, survival as well as the generation of immunological memory by controlling the transcriptional activity of several targets including PI3K-AKT and NF-κB. Beyond the well-established activator role in effector T cells, OX-40 is also expressed by regulatory T cells where, it plays as a Forkhead box P3 (FoxP3) inhibitor thus preventing Treg activation and *de novo* generation (6–8). It has been recently demonstrated that costimulatory activity of OX-40 can also improve NK cell activation and cytokine production, enhancing their cytotoxicity against cancer cells (9), even though there is a lack of information on the role and engagement of OX-40 in NK cells.

Given its role in the immune response, OX-40 is the target of several drugs in progress for immune-mediated diseases (10). Agonists or antagonists of OX-40 are being developed to be respectively implemented for therapy of cancer or auto-immune diseases. A few monoclonal antibodies targeting OX-40 have already been generated and are currently in clinical trials. Examples are represented by Telazorlimab and Rocatinlimab, two anti-OX-40 monoclonal antibodies able to prevent T cell activation and proliferation and used to treat atopic dermatitis (11). It is also known that OX-40 promotes T cell survival and sustains the humoral response during viral infections (12). The anti-viral effects of OX-40 agonistic mAbs has been studied in patients affected with chronic hepatitis B where OX-40 is crucial to suppress HBV replication, by stimulating the activity of CD8+ T cells (13).

As mentioned above, OX-40 activation is an attractive field in cancer immunotherapy due to its dual capability to stimulate antitumor effector T cells and to dampen Treg function. The treatment with OX-40 agonists promotes T cell activation and infiltration into tumors and it also prevents primary or acquired resistance to drugs targeting inhibitory checkpoints (e.g., PD-1, CTLA-4) as depicted in different preclinical cancer models (B16 melanoma, Lewis lung carcinoma, colon cancer and 4T-1 breast cancer). Beyond functional invalidation of Treg cells, if of appropriate isotype, anti-OX-40 mAbs can further eliminate infiltrating Treg by Antibody-Dependent Cellular Cytotoxicity (ADCC), as reported for several malignancies such as MCA303 sarcoma, CT26 colon carcinoma and SM1 breast cancer (14, 15).

Several anti-OX-40 agonistic antibodies are currently being tested in clinical trials, both in monotherapy and in combination with other drugs (16). As a monotherapy, the first in-human phase I clinical trial (NCT01644968) evaluated the potential therapeutic effect of MEDI6469, a mouse IgG1 agonistic mAb anti-OX-40, in metastatic solid malignancies. However, the elicitation of human anti-mouse antibodies induced by the murine mAb hampered its therapeutic potential (17, 18). For this reason, a humanized version of the antibody, MEDI0562, was tested in a phase I study (NCT02318394) in advanced solid tumours, mostly head and neck squamous cell carcinoma demonstrating its safety even though showing limited antitumor efficacy as monotherapy (19). Therefore, to overcome this limitation, combinatorial treatments are still being considered in ongoing clinical trials, especially with antiPD-1 and anti-PD-L1 mAbs (20). Blocking PD-1\PD-L1 axis, which heavily inhibits T cell expansion, with simultaneous stimulation of OX-40 has proven to be a successful approach. The combination of anti–PD-1 antagonistic and anti-OX-40 agonistic mAbs was notably effective in several aggressive and poorly immunogenic tumors, such as ovarian and pancreatic cancer (21, 22). Prominent examples of clinically successful anti-OX-40 mAbs are represented by clinical studies of GSK3174998, a humanized anti-OX-40 IgG1 under evaluation in combination with the anti-PD-1 Pembrolizumab (NCT02528357), and by the phase I/II trial of INBRX-106, another anti-OX-40 agonistic antibody with Pembrolizumab (NCT04198766) (23). Despite the interesting results emerging from these clinical trials, there is still a strong need of novel human anti-OX-40 mAbs due to unclear mechanisms of action of current antibodies and impact of recognized epitope on T-cell activation, OX-40 clustering and signalling. In our laboratory, we previously identified human anti-OX-40 scFvs by phage display selections on both human activated lymphocytes and recombinant OX-40 protein used as baits to favor the isolation of mAbs recognizing the target in its native configuration (24–26). In this study, we converted these scFvs into full IgG1 human mAbs, named OX-40_1, OX-40_2, OX-40_3, and OX-40_5, and we extensively characterized them both for their binding to OX-40 and for their ability to compete with the natural ligand (OX-40-L). Moreover, they were tested for their biological effects on cultures of human peripheral blood lymphocytes and on co-cultures of hPBMCs and tumor cells or NK and Treg cells, in order to evaluate their potential as new therapeutic tools. We also took advantage of the novel mAbs to clarify the role of OX-40 on NK cells.

## 2 Materials and methods

### 2.1 Antibodies and human recombinant proteins

The following human recombinant (hr) proteins were used:

OX40/TNFRSF4 Fc Chimeric Protein, CF (3388-OX), human OX40 Ligand/TNFSF4 Protein, Fc Tag (OXL-H5266) from Acro biosystems (Newark, DE 19711, USA), Recombinant Human IgG1 Fc Protein, CF (110-HG), Recombinant Human OX40/TNFRSF4 His-tagged Protein (9969-OX), CF, all from R&D SYSTEMS Inc. Recombinant Human OX40/TNFRSF4 Protein (hFc & AVI Tag), biotinylated 10481-H41H-B purchased from Sino Biological. OX40L (CD252), His-Tag recombinant (SRP0571) by Sigma-Aldrich.

The human commercial or clinically validated antibodies used are reported as follows:

CD134/OX40 Polyclonal antibody (20006-1-AP) from Proteintech; Goat anti-Rabbit IgG (H+L) Secondary Antibody, HRP (31460, Invitrogen, Thermo Fisher Scientific); anti-human IgG (Fab’)2 HRP-conjugated goat monoclonal antibody (ab87422, Abcam, Cambridge, UK); anti-alpha Tubulin antibody (ab4074, Abcam); anti-vinculin monoclonal antibody (sc-25336, Santa Cruz Biotechnology, Inc. Dallas, TX, USA) HRP-conjugated anti-mouse IgG secondary antibody (Sigma-Aldrich). The clinically validated anti-PD-L1 Atezolizumab mAb (N298A, InvivoGen) and anti-OX-40 Rocatinlimab (HY-P99955, MedChemExpress) were also used.

### 2.2 Cell cultures

The human triple-negative breast cancer (TNBC) MDA-MB-231 cells were obtained from the American Type Culture Collection (ATCC), and cultured in Dulbecco’s Modified Eagle’s Medium (DMEM, Gibco, Life Technologies). The medium was supplemented with 10% (vol/vol) heat-inactivated fetal bovine serum (30-2025, ATCC), 50 U/mL penicillin, 50 µg/mL streptomycin, and 2 mM L-glutamine (all from Gibco Life Technologies) and cultured in a humidified atmosphere of 95% air and 5% CO_2_ at 37°C.

### 2.3 Production and Purification of the novel antibodies

Plasmid vectors encoding each of the 5 novel anti-OX-40 mAbs were transfected into HEK293-EBNA SINEUP cells (25) plated in 150 mm Corning^®^ tissue-culture treated culture dishes. Aliquots of 20µL of Lipofectamine^®^ 2000 (11668019, Invitrogen, Thermo Fisher Scientific) and 10 µg of DNA vectors (2:1 ratio) were diluted, for each construct, in serum free-DMEM high glucose, pyruvate (41966-029, Gibco, Life Technologies). The plates were washed using the same medium supplemented with 50U/mL penicillin, 50μg/mL streptomycin, and then the mixtures containing DNA-lipid complexes were added to each plate. After 5 hours of incubation at 37°C, cell culture media was replaced with chemical defined (CD) CHO medium (10743-011, Gibco, Life Technologies) supplemented with 50U/mL penicillin, 50μg/mL streptomycin and 2 mM L-glutamine (25030-024, Gibco, Life Technologies). After 10 days of incubation at 37°C, the supernatants were collected, centrifuged at 2000g for 20 min. The antibodies were purified from the CD medium by using affinity chromatography Protein A HP SpinTrap columns (28903132, Cytiva, by Global Life Sciences Solutions LLC), according to manufacturer’s instructions. The purified monoclonal antibodies were analyzed under reducing conditions by SDS-PAGE and Coomassie Staining analysis, as previously described (24), to evaluate the purity and protein aggregation, then they were aliquoted and stored either at 4°C or at -80°C. In order to analyse the stability after one month storage in different conditions, aliquots were tested in parallel by Western Blotting and ELISA assays at two concentrations (10, 100 nM) on immobilized OX-40/Fc recombinant protein (5 µg/ml).

### 2.4 Enzyme-linked immunosorbent assays

#### 2.4.1 ELISA on purified protein

In order to evaluate the binding affinity of the novel OX-40 mAbs, parallel ELISA assays were performed on human OX-40/Fc recombinant protein and, as a negative control, on recombinant human IgG1 Fc protein. The proteins were coated at a concentration of 5 µg/ml. The anti-OX-40 mAbs were incubated at Room Temperature (RT) for 1 h 30 min at increasing concentrations (0.3–200 nM) with gentle shaking. After three extensive washes with PBS 1X, the secondary anti-human HRP-conjugated IgG (Fab’)2, was added for 1 h at RT. The plate was washed three times with PBS 1X and the signal was detected by incubating with 3,3’,5,5’-Tetramethylbenzidine (TMB) (Sigma-Aldrich) reagent before quenching with an equal volume of 1 N HCl. Finally, the absorbance at 450 nm was measured by Envision plate reader (Perkin Elmer, 2102). The binding values were reported in GraphPad Prism 10 tool system for the binding curve analyses and the K_D_ values calculation according to the model: Y = Bmax*X/(Kd+X) + NS*X + Background.

#### 2.4.2 Cell ELISA on human PBMCs

The binding of the purified anti-OX-40 mAbs to the target, expressed in its native conformation, was tested on human lymphocytes. First, the expression levels of OX-40 on hPBMCs, either untreated or activated with SEB (50 ng/ml), was checked by plating 2.10^5^ cells/well and incubating them for 1h and 30 min with a commercial CD134/OX40 Polyclonal antibody. The signal was detected by incubating the cells with the secondary HRP-conjugated anti-Rabbit IgG (H+L).After extensive washes, 3,3’,5,5’-Tetramethylbenzidine (TMB) was added. After quenching with HCl 1N, the plate was read at 450 nm by Envision plate reader.

The binding curves of the novel anti-OX-40 mAbs were obtained by incubating them at increasing concentrations (ranging from 0.3 to 200 nM) for 1 h and 30 min at RT, on both untreated and activated lymphocytes, plated as mentioned above. After three washes, the secondary anti-human HRP-conjugated IgG (Fab’)2 was incubated for 1h at RT and the signal was measured as previously mentioned. The binding of OX-40_5 mAb was also tested on CD4^+^/CD25^-^ or Treg cells isolated from hPBMCs and stimulated with SEB for 48 h. Cells were plated at a density of 2.10^5^ cells/well, incubated with OX-40_5 (150 nM) for 1 h and 30 min at RT and the signal detected by using the secondary anti-human HRP-conjugated IgG (Fab’)2 as previously mentioned.

#### 2.4.3 Competitive ELISA assays

Competitive parallel ELISA assays were carried out in order to determine whether the novel mAbs recognize different epitopes. To this aim, OX-40_2 and OX-40_1 were immobilized on 96-well plate (5 µg/mL in a buffer of NaHCO_3_). After 48 h, the recombinant biotinylated OX-40 protein was pre-incubated in the absence or presence of the other mAbs in 3% BSA/PBS at the saturating concentration of 250 nM (5-fold molar excess) in agitation for 1 h at RT. As a negative control, the receptor was also pre-incubated with the same mAb used for the coating at the same saturating concentration. The preincubation mixtures were added to the plate for 1 h 30 min and after extensive washes with PBS, the binding was detected by incubation with HRP-conjugated streptavidin (710005, Bio-Rad, Hercules) for 30 min and measured by following the ELISA procedure mentioned above.

### 2.5 Biolayer interferometry analyses

#### 2.5.1 Binding kinetics via BLI analyses

In order to analyze the kinetics of the four purified anti-OX-40 mAbs, BLI analyses were performed by using the Octet R4 Protein Analysis System (Sartorius, Fremont, CA, USA) by following the manufacturer’s recommendation, as previously reported (27, 28). Briefly, after hydrating the Pro-A biosensors for 15 min with kinetic buffer (KB) 10×, the latter were loaded with OX-40 Fc recombinant protein (from R&D Systems) at a concentration of 3 µg/ml. Since the analytes contained Fc portion, the biosensors were saturated to avoid non-specific interactions of the mAbs to the proA biosensors by a second load with the human Fc recombinant chimeric protein (from R&D Systems). After the loading, the association step was carried out by incubating the biosensors up to 500 s in a solution containing the anti-OX-40 mAbs diluted in KB buffer 10× at increasing concentrations (10, 50 and 100 nM). The dissociation step was performed in KB buffer 10 × for 250 s and finally the biosensors were regenerated in a solution of HCl 1N. The data were obtained and processed into the Octet Analysis Studio Software 13.0.{it}.

#### 2.5.2 Epitope binning via BLI in tandem assay

For epitope binning analyses, the loading step was carried out as described above. After washing step, OX-40-Fc, used as ligand, was saturated with the first mAb (OX-40_2 or OX-40_5) at concentration of 200 nM for 600 s; then, the second mAb (OX-40_3 or OX-40_1) was added at increasing concentrations (50 nM, 100 nM, and 200 nM) for 600 s. Alternatively, in additional analyses, Rocatinlimab was used as first Ab for saturation, and each of the novel mAbs was added at a concentration of 200 nM. The biosensors were regenerated and the data acquired and processed as mentioned above.

### 2.5.3 Competitive BLI assays

In order to evaluate the interference of the novel isolated mAbs in the binding between the OX-40 Ligand and OX-40 Receptor, we performed competitive BLI assays. OX-40L/His ligand was immobilized on HIS1K biosensors at a concentration of 5 μg/mL; after washes, OX-40/Fc receptor was added for 500 s at a concentration of 10 nM alone or previously incubated with one of each anti-OX-40 mAb (10-fold molar excess) for 1 h 30 min at RT. A parallel pre-incubation with an unrelated IgG1 mAb was carried out as a negative control. The data were obtained and processed by the Octet Analysis Studio Software 13.0{it}.

### 2.6 Bioluminescent cell-based assays

In order to test whether the antibodies can activate OX40 downstream pathway, the bioluminescent OX40 Bioassay (JA2191, Promega) was used. Briefly, the day before the assay, the Jurkat T cells expressing OX40 were thawed in RPMI with 5% FBS and plated in white, flat-bottom plate by following the manufacturer’s recommendations. After 24 h, the five novel anti-OX-40 mAbs or the ligand, used as a positive control, were added at increasing concentrations (1.5–200 nM) and incubated for 5 h in 5% CO_2_ incubator at 37 °C. After adding the Bio-Glo Reagent to the wells for 5–15 min, the signal was measured by using a luminescence reader (Envision plate reader by Perkin Elmer).

### 2.7 Isolation of Human Peripheral Blood Mononuclear Cells (hPBMCs)

Human PBMCs were purchased from ATCC (PCS-800-011). All tissues used for isolation by ATCC are obtained under informed consent and conform to HIPAA regulations to protect the privacy of the donor’s Personally Identifiable Information. The vials containing the PBMCs were gently thawed out by using a medium composed by RPMI 1640 with 1% L-glutamine, 1% CTLWash™ (Immunospot by Cellular Technology Limited) and 100 U/mL Benzonase (Merck Millipore) and used after resting overnight at 37°C in R10 medium (RPMI 1640 supplemented with 10% inactivated FBS, 1% L-glutamine, 50 U/mL penicillin, 50 µg/mL streptomycin and 1% HEPES all from (Gibco, Life Technologies).

### 2.8 Isolation of NK and CD8^+^ T cell

Human NK and CD8^+^ T cells were isolated from unfractioned hPBMCs by using NK Cell (130–092–657) or CD8^+^ T Cell Isolation Kit (130–096–495), both from MACS by Miltenyi Biotec, as previously reported (29, 30), according to the manufacturer’s guidelines for the magnetic labelling. First, lymphocytes were incubated with NK or CD8^+^ T Cell Biotin-Antibody Cocktail for 5 min at 4°C, and then they were incubated with NK or CD8^+^ T Cell MicroBead Cocktail for 10 min at 4 °C. After placing the tube in the magnetic field of a suitable separator, the NK cells or CD8^+^ T Cell in the supernatants were collected and then resuspended in R10 medium.

### 2.9 Isolation of CD4^+^ T cells or CD4^+^/CD25^+^ regulatory T cells

CD4^+^/CD25^+^ regulatory T cells were isolated from non-fractioned hPBMCs by using the Dynabeads Regulatory CD4^+^/CD25^+^ T Cell Kit (Invitrogen by Thermo Fisher Scientific), following the manufacturer’s instructions. Briefly, starting from PBMCs, the non CD4^+^ T cells were labelled with the Antibody Mix Human CD4 provided by the kit and then incubated with the Depletion MyOne Dynabeads for their removal. The CD4^+^ T cells in the supernatant were either collected and resuspended in R10 medium for immediate use or further incubated with the Dynabeads CD25, to obtain the pool of CD4^+^/CD25^+^ T cells. After the incubation with Dynabeads, the cells were placed on a magnetic stand to remove the supernatant containing CD4^+^/CD25**^-^** T cells. The bound cells were eluted by incubating with the DETACHaBEAD reagent after extensive washes, and collected by placing the sample on a magnetic separator in order to remove the Dynabeads. The supernatant containing the CD4^+^/CD25^+^ isolated Treg cells was centrifuged at 350 × *g* for 8 min and the cell pellet was then resuspended in R10 medium.

### 2.10 ADCC assays and determination of cell lysis

To clarify whether the novel anti-OX-40 mAbs could induce Treg and tumor cells killing through ADCC mediated by NK cells, the latter isolated cells (6 × 10^4^) were co-cultured with CD4^+^/CD25^+^ regulatory T cells, or with control CD4^+^/CD25^-^ T cells, or with MDA-MB-231 TNBC cell line (Effector:target ratio 3:1) in the presence of OX-40_1, _2, _3 or_5, tested at the concentrations of 1 nM and 10 nM, or 100 nM, respectively. As a negative control, the co-cultures were incubated also in the absence or presence of an unrelated IgG1. The ADCC induced by NK cells was evaluated by measuring the Lactate DeHydrogenase (LDH) released in the medium of co-cultures after 48 or 72 h of treatment (29), by using the LDH detection Kit (Thermofisher Scientific). In order to further confirm the results obtained from cytotoxic assay, the IFNγ secretion was evaluated in the supernatants of co-cultures by following manufacturer’s instructions provided by the kit “Human IFN-gamma DuoSet ELISA” (DY285B, R&D Systems) and fully described in the corresponding section “Cytokines secretion assay”.

### 2.11 Cytokine secretion assays

The supernatants were analyzed by performing ELISA assays in order to evaluate the levels of IL-2 and IFNγ secretion. The IL-2 (DY202) and IFNγ (DY285B) DuoSet ELISA kits by R&D Systems were used to measure the release of cytokines from cell culture supernatants. The assays were performed as described (30, 31) and the absorbance values were converted in pg/mL, according to the producer’s instructions, by plotting the mean absorbance for each sample in comparison with the standard curve constructed within the experiment. Concentration values were reported as the means of at least three determinations.

### 2.12 Cytotoxicity assays

The cytotoxic effects of the novel anti-OX-40 mAbs were tested in co-cultures of hPBMCs and MDA-MB-231, either used as single agents or in combination with the clinically validated Atezolizumab. To this aim, MDA-MB-231 were plated at a density of 1 × 10^4^ cells/well, in 96-well flat-bottom plates and incubated overnight at 37°C; then, the lymphocytes were added (5:1 effector/target ratio) and the co-cultures were treated with each mAbs or their combinations at the concentration of 5 µg/mL for 48 h at 37°C. As negative controls, the cytotoxic effects on co-cultures were analyzed, in parallel, in the absence of treatments or in the presence of an unrelated IgG1 used in the same conditions. After 48 h the supernatants were collected, and the levels of LDH released by the cells was evaluated and expressed as a percentage with respect to the max lysis obtained after treatment with 10% Triton X-100, used as a positive control, following the manufacturer’s recommendations for CyQUANT™ LDH Cytotoxicity Assay (Thermofisher Scientific).

### 2.13 Western blotting analyses

In order to evaluate the OX-40 expression in different lymphocytes subpopulations or other non-immune cells, Western Blotting analyses were carried out. Untreated lymphocytes or activated total hPBMCs, NK, CD8^+^ T, CD4^+^ T and Treg cells, previously isolated as described above, were plated at a density of 1×10^6^ cells/well in 48-well flat-bottom plates and stimulated for 72 h with SEB (50 ng/mL). Then, the cell pellets obtained were resuspended in lysis buffer containing 10 mM Tris-HCl pH 7.4, 0.5% Nonidet-P-40, 150 mM NaCl, 1 mM Sodium orthovanadate (Sigma-Aldrich) and protease inhibitors (Roche). After 20 minutes, when the lysis was completed, the protein concentration of cell extracts was determined with the Bradford colorimetric assay (Sigma-Aldrich). Western Blotting analysis was performed by incubating the nitrocellulose filter with the commercial anti-OX40 Polyclonal antibody, human OX-40 Ligand trimeric Fc-tagged, OX-40_3 or OX-40_5 followed by the secondary HRP-conjugated anti-human IgG (Fc-specific) or HRP-conjugated anti-Rabbit IgG (H+L). The protein levels are expressed as fold increase with respect to untreated hPBMCs and the intensity of the bands was normalized to either tubulin or vinculin by using Image Lab software 6.0.1, and by calculating the ratio of OX-40/loading control signal intensities for each cell extract.

### 2.14 Data analysis and statistics

Error bars were calculated on the basis of the results obtained by at least three independent experiments. Statistical analyses were assessed by Student’s t-test (two variables) or by Sidak’s multiple comparisons test performed after Two-way ANOVA analysis. Statistical significance was established as *** p ≤ 0.001; ** p < 0.01; * p < 0.05.

## 3 Results

### 3.1 Phage display selection of human scFvs specific for OX-40 and screening by NGS

In our laboratory a human antibody repertoire against several immune checkpoints was previously generated (24–26), by performing a new strategy of phage display selection based on coupling panning cycles on human activated lymphocytes expressing the targets in their native conformation with cycles performed on recombinant target proteins to enrich specific binders.

As resting lymphocytes do not express OX-40, the first step of selection included a negative panning on non-activated (resting) human lymphocytes to subtract from the phage pool those recognizing common antigens on cell surface, followed by a positive selection on activated lymphocytes expressing high levels of OX-40 (24). The recovered phages were amplified in *E. coli* TG1 cells for the following selection step to be performed on recombinant chimeric OX-40/Fc.To this aim, as recombinant protein was fused to Fc fragment, as shown in **Supplementary Figure S1**, the amplified phages were submitted to two following rounds of negative panning by incubating them with human recombinant IgG1-Fc coated protein. The unbound phages in the supernatant were then incubated with rhOX-40/Fc protein immobilized in another tube for positive selection. The bound phages were then eluted, and phagemid DNA was prepared for next generation sequencing to in silico predict the binding of phages based on enrichment kinetics of given sequences during selection cycles (see **Table 1**). Due to limitation in fragment size evaluation by NGS platforms, the sequencing was focused on the VH fragments by paired-ends 2 × 300 on the Illumina MiSeq platform. As previously reported (24, 26), the OX-40 screening was very productive by showing a significant enrichment of multiple consensus sequences. As phage panning selects for binding property of protein rather than nucleotide sequence, the identification of different clones with synonymous nucleotide sequences represents the best valuable feedback of a productive selection. Only 10% of the VHs identified in cycle 3 sub-library contained stop codons or frameshift indels in complementary determining or framework regions; these sequences were discarded from further analysis. The resulting sub-library was interpolated with a “target unrelated database” which consists of a repository of VHs sequences identified from many different screenings performed during the years against different targets (26). This database is hypothesized to contain sequences of clones binding to the common baits or tags of recombinant proteins (e.g., Fc region of Fc-fused recombinant proteins used for phage library panning) or plastic polymers. This funnel filtering allowed to discard an additional 40% of clones from cycle 3 sub-library, allowing us to further restrict the analysis of potential binders to 50% of VHs. Top ranking clones (most enriched ones) were physically rescued by inverse PCR to identify those VL chains linked to the given VHs of interest. Full scFv sequences allowed us to identify redundant clones made of degenerate synonymous nucleotide sequences. We thus focused our analysis on non-synonymous clones 1, 2, 3, 5 and 8 (**Table 1**). Light chains resulted respectively lambda for 1, 2 and 3 and kappa for 5 and 8. The scFvs were converted into fully human monoclonal antibodies by sub-cloning the variable domains in frame to human IgG1 Fc into mammalian expression vectors as previously described (24).

**Table 1:** Enrichment of the scFv sequences over the 3 phage display selection cycles.

Clone	Cpm cycle_2	Cpm cycle_3	Cycle_3 fraction occupancy (%)
OX-40_1	6,90E+04	5,36E+05	61
OX-40_2	1,70E+04	4,20E+04	5
OX-40_3	4,95E+03	3,39E+04	4
OX-40_5	6,38E+03	1,86E+04	2
OX-40_8	2,13E+03	4,50E+03	0,5

The table reports the relative sequences enrichments over the three selection cycles.

### 3.2 Production, purification and analysis of stability of the novel anti-OX-40 mAbs

Expression plasmids encoding each of the 5 novel anti-OX40 mAbs were transfected into HEK293-EBNA cells for 10-days semi-stable production of recombinant antibodies. The secreted mAbs were purified from conditioned medium by protein A affinity chromatography as described in Methods. The purified antibodies were tested for their purity and stability by SDS-PAGE, Western Blotting and ELISA assays. As shown in **Supplementary Figure S2**, they showed the expected Molecular Weights and no contaminants or degradation products were detected by SDS-PAGE. A preliminary stability test was also performed to assess the effects of 30 days storage at 4 °C or after one cycle of freezing and thawing out at -80 °C. We found that all the novel mAbs retained their concentration, structural stability and binding properties in both conditions (see **Supplementary Figures S3A, B**), thus suggesting that storage at 4 °C for 1 month or a cycle of freezing and thawing out does not significantly affect their stability. A slight loss of binding was observed only with OX_40_2 after a cycle of freezing and thawing out. The mAb OX-40_8 did not bind either at time 0 and was used as a negative control. Moreover, to confirm the binding specificity of the novel isolated mAbs for OX-40, WB analyses were performed by incubating the membrane, loaded with human OX-40/His recombinant protein, with OX-40_3 or OX-40_5 mAbs, in parallel with a commercial anti-OX-40 polyclonal Ab. As shown in **Supplementary Figure S3C**, the commercial antibody detected two glycosylated forms of OX-40 recombinant purified protein. Similarly, the novel mAbs recognized both protein forms, but OX-40_5 mAb showed a preferential binding to the medium glycosylated form of OX-40. These results confirm the binding specificity of the novel mAbs for OX40.

### 3.3 Characterization of binding ability of the novel converted anti−OX-40 mAbs by ELISA and Biolayer Interferometry

**Figure 1:**
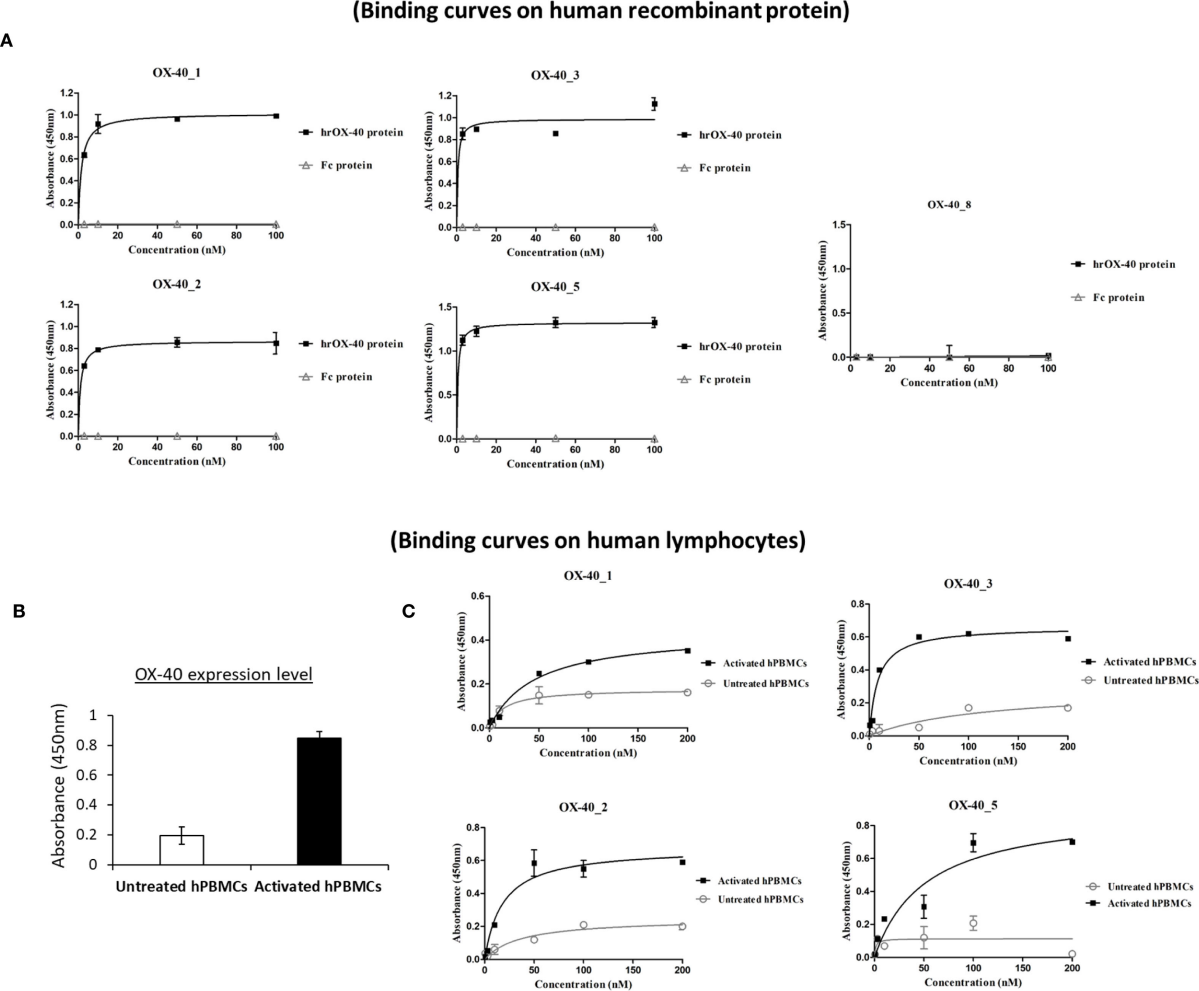
Binding of the novel anti-OX-40 mAbs on OX-40 recombinant protein and human lymphocytes. **(A)** Binding of the novel human mAbs was tested at increasing concentrations (0.3–100 nM) on purified rhOX-40 and the signal was detected by using the secondary HRP-conjugated anti-Fab antibody. OX-40_8 mAb was used as a negative control. **(B)** The expression level of OX-40 protein was determined on untreated or activated hPBMCs by using a commercial polyclonal anti-OX-40 Ab. **(C)** Binding of mAbs to untreated (grey curves) and activated (black curves) hPBMCs tested by cell ELISA at increasing concentrations (0.3–200 nM). Concentration values were reported as the mean of at least three determinations and error bars depicted means ± SD. The binding curves were obtained by using Prism (GraphPad Prism 5) tool. The results were obtained by at least three independent experiments.

The newly-generated anti-OX-40 mAbs were further tested for their binding to the targets, by performing a dose-response binding curve by ELISA assays (0.3–200 nM) on human OX-40 expressed either as recombinant protein or in its native conformation on activated lymphocytes. The binding curves, shown in **Figure 1A**, demonstrate that 4 out of 5 antibodies bind with high affinity (nanomolar or subnanomolar range) to human recombinant OX-40/Fc protein, whereas no binding was detected on isolated Fc used as a negative control, thus suggesting their binding specificity for the target. Also in these assays, OX-40_8 mAb did not show again a significant binding for OX-40 receptor and was discarded for the following analyses.

In order to evaluate the binding of OX-40_1, 2, 3 and 5 mAbs to the target also when expressed on human peripheral blood lymphocytes (hPBMCs), we first checked the expression levels of OX-40 on hPBMCs, either untreated or stimulated with Staphylococcal enterotoxin B (SEB), by using a commercial anti-OX-40 Ab for detection. As reported in **Figure 1B**, we observed a significant increase in OX-40 expression 72 h after stimulation. Untreated hPBMCs were used as a negative control, due to the lack of OX-40 expression (7). Thus, we performed parallel binding curves by testing the novel mAbs at increasing concentrations (0.3-100 nM), either on resting or SEB-activated lymphocytes. As shown in **Figure 1C**, all the novel antibodies were able to bind to the native target displayed by activated lymphocytes in a nanomolar range, whereas a very poor binding was detected on untreated hPBMCs reinforcing evidences of their specificity for OX-40.

**Figure 2:**
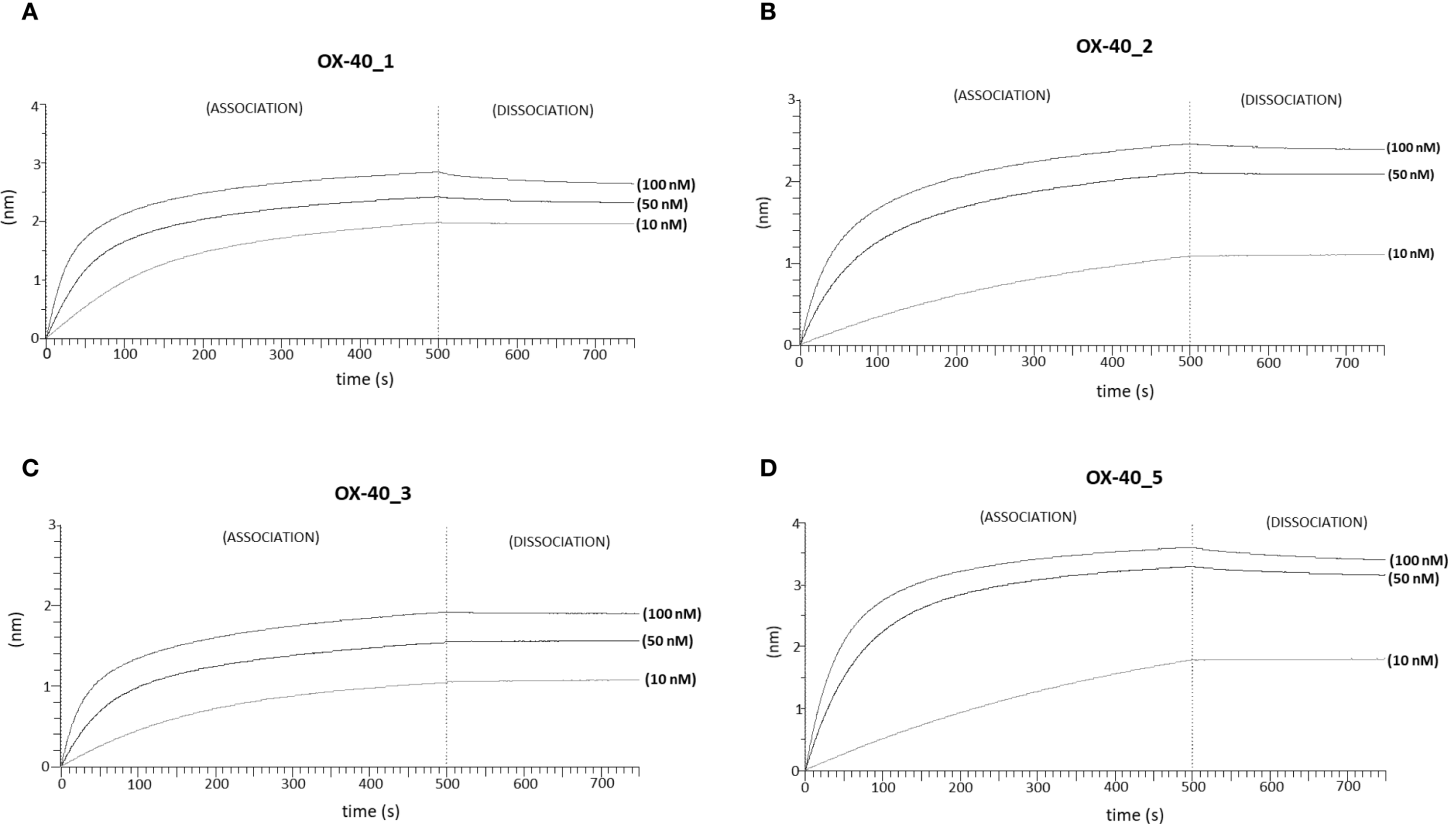
Binding kinetics of the novel anti-OX-40 mAbs on immobilized OX-40 protein via BLI analyses. The sensorgrams report the binding of novel converted mAbs to OX-40 obtained via BLI analyses. The recombinant human OX-40/Fc protein was used as a ligand and immobilized on ProA sensor (3 µg/mL), whereas OX-40_1 **(A)**, OX-40_2 **(B)**, OX-40_3 **(C)** and OX-40_5 **(D)** were used as analytes and tested at increasing concentrations (10–100 nM), after saturation with Fc protein by two subsequent injections (6 µg/mL). The sensorgrams show association and dissociation rates of the analytes.

We further analysed the binding kinetics (association and dissociation rates not detectable by ELISA) of the four functional anti-OX-40 mAbs by carrying out real time binding analyses by Biolayer Interferometry (BLI). In these assays, we immobilized by protein A the human recombinant OX-40/Fc on the surface of the biosensors. Before adding the analytes, we further saturated the biosensors by using the recombinant human Fc to avoid non-specific interactions of the antibodies with the proA biosensor through their Fc fragment. Then, we added each of the four anti-OX-40 antibodies, used as analytes at increasing concentrations (10, 50 and 100 nM). These experimental conditions, in which the target protein was immobilized on the biosensor, were chosen to mimic the *in vivo* environment, where the circulating bivalent antibodies could bind to the targets exposed on the surface of immune cells. As shown in **Figure 2**, all the antibodies bound to their targets in a dose-dependent fashion with K_D_ values in a nanomolar range. The relative K_D_ values are reported in **Table 2**, with association and dissociation constants. OX-40_3 was found to have a K_D_ in a nanomolar range with a dissociation rate one order of magnitude slower than those of the other mAbs. Similar results were obtained for OX-40_1, _2, _3 when BLI assays were repeated by immobilizing OX-40/his on HIS1K biosensors and by adding the novel mAbs as analytes. The affinity of OX-40_2 resulted slightly lower in the latter system likely due to the different conformation between OX-40/Fc and OX-40/HIS fusion proteins. In **Table 3** a comparison of the K_D_ obtained by BLI with those obtained by ELISA on purified protein and by cell ELISA on activated hPBMCs are reported. K_D_ values extrapolated by cell ELISA were found higher than those obtained on the purified protein, but again, OX-40_3 showed the best properties followed by OX-40_2, whereas OX-40_1 and OX-40_5 lost some affinity for the receptor on cell surface.

**Table 2:** Binding affinities and kinetics of the novel mAbs to immobilized OX-40/Fc recombinant protein by BLI.

Mab	K_D_ (M)	K_D_ Error	Ka (1/ms)	Kdis (1/s)
OX-40_1	2.67 x10^-9^	8.17 x10^-11^	1.45 x10^5^	2.93 x10^-4^
OX-40_2	6.46 x10^-9^	7.61 x10^-9^	4.52 x10^4^	1.48 x10^-4^
OX-40_3	1.40 x10^-9^	1.30 x10^-9^	7.42 x10^4^	9.07 x10^-5^
OX-40_5	3.81 x10^-9^	3.23 x10^-11^	9.78 x10^4^	2.80 x10^-4^

The table reports K_D_ values (M) with association and dissociation rate constants of the novel mAbs tested by BLI analyses by using proA biosensors. Analysis was performed using Octet Analysis Studio 13.0 Software (Sartorius, Fremont, CA, USA) (27).

**Table 3:** Binding affinities of the novel mAbs to immobilized OX-40 recombinant proteins and human lymphocytes by ELISA compared to those measured by BLI.

Mab	K_D_ (by BLI) (rOX-40/Fc)	K_D_ (by ELISA) on OX-40/Fc	K_D_ (by cell ELISA) on activated hPBMCs	K_D_ (by BLI) (rOX-40/his)
OX-40_1	2.67 nM	1.6 nM	45.5 nM	7.49 nM
OX-40_2	6.46 nM	1 nM	18 nM	13 nM
OX-40_3	1.40 nM	0.56 nM	8.5 nM	1.92 nM
OX-40_5	3.81 nM	0.57 nM	52 nM	4.09 nM

The table reports a comparison of the K_D_ values (nM) of the indicated antibodies obtained by measuring the binding, via BLI analyses (both proA biosensor and HIS1K biosensors) or by ELISA assays, either on purified recombinant OX-40 or expressed on activated hPBMCs surface.

### 3.4 Investigation on the epitopes recognized by the novel anti-OX-40 mAbs via BLI and ELISA assays

**Figure 3:**
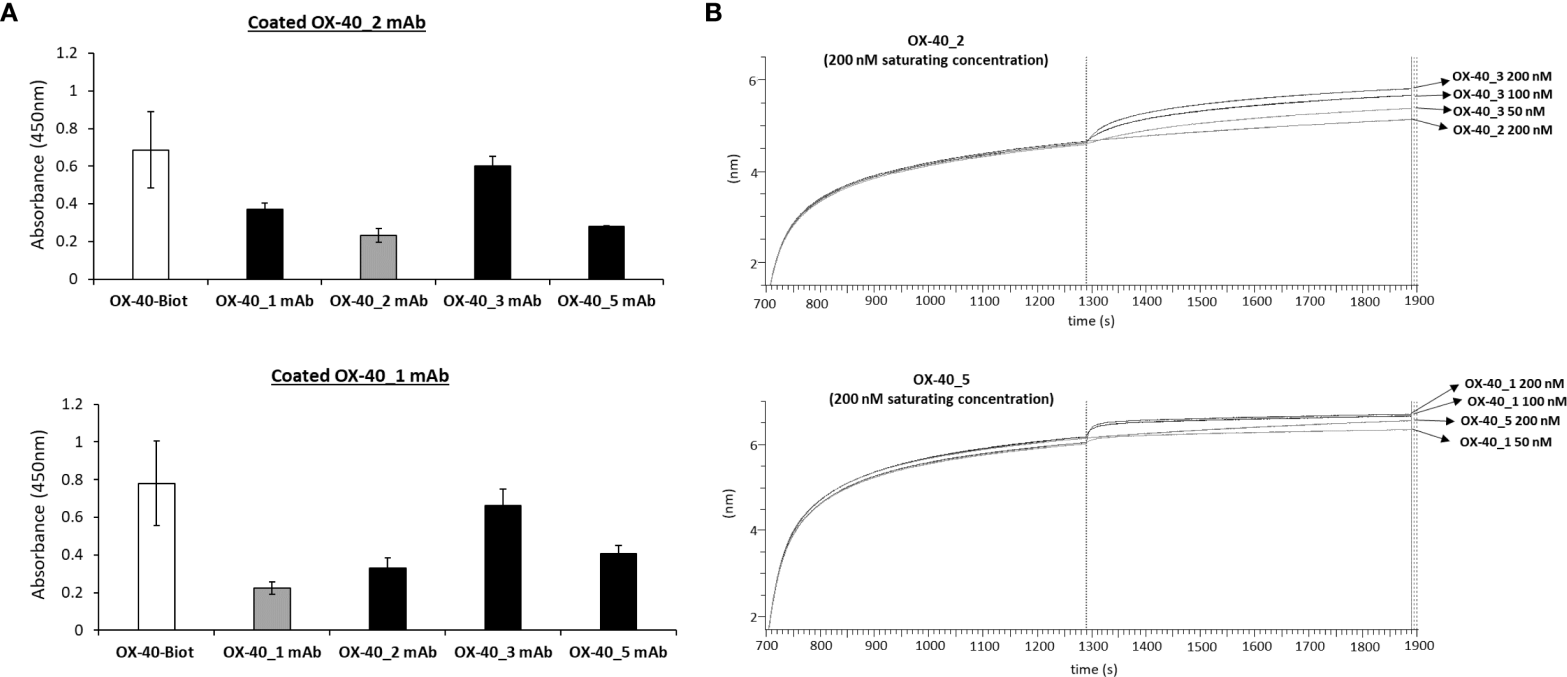
Epitope binning of the novel mAbs on OX-40 receptor. **(A)** ELISA assays were performed by immobilizing OX40_1 or OX-40_2 mAb at the concentration of 30 nM, then OX-40/Fc biotinylated protein was added to the coated plates after preincubation of 1 h with a 5-fold molar excess of each of the other mAbs (black bars). A preincubation of receptor with the same immobilized mAb was used as a control (grey bars). **(B)** BLI in tandem assays were performed by immobilizing OX40/Fc protein on proA biosensor (20 nM) and saturating with OX-40_5 (200 nM) or OX-40_2 mAb (200 nM). The binding of the analyte OX-40_1 or OX-40_3 mAb, respectively was then measured at increasing concentrations (50–200 nM). The results were obtained by at least three independent experiments. Error bars depicted means ± SD.

Once validated the binding ability of the novel anti-OX-40 mAbs, we investigated whether they recognize different regions on their target by epitope binning analyses by ELISA and BLI assays. To this aim, each of the novel binders was tested in competitive assays by measuring the binding of biotinylated OX-40 receptor to the immobilized mAb after an incubation of 1 h at RT in the absence or presence of saturating concentration (250 nM) of the other mAb. The signal was detected by using HRP conjugated Streptavidin. As a negative control, the binding was also evaluated when the receptor was pre-incubated with saturating concentrations of the same mAb used for the coating. As shown in **Figure 3A**, the binding of OX-40 receptor to OX-40_2 was not affected by OX-40_3 and only partially affected by OX-40_1 and OX-40_5. The binding of the receptor to OX-40_1 was not affected by OX-40_3 and partially affected by OX-40_2 and OX-40_5.

To further confirm these findings, additional epitope binning analyses were carried out via BLI by using a different in-tandem approach, with respect to the previous premix assays. Briefly, recombinant OX-40/Fc receptor was immobilized on ProA biosensors, then a first antibody (either OX-40_5 or OX-40_2) was added at saturating concentrations of 200 nM, and the second antibody (OX-40_1 or OX-40_3, respectively) was added at increasing concentrations (50–200 nM). The results, reported by the sensorgrams in **Figure 3B**, showed that OX-40_1 and _3 are still able to bind to the protein even after the saturation with OX-40_5 or _2, therefore confirming the results observed by ELISA assays. Altogether, these results suggest that these antibodies belong to different family bins and thus recognize distinct or only partially overlapping epitopes. Since the epitopes can significantly impact on their biological effects, it is an advantage getting a collection of mAbs recognizing different epitopes that could be potentially combined.

**Figure 4:**
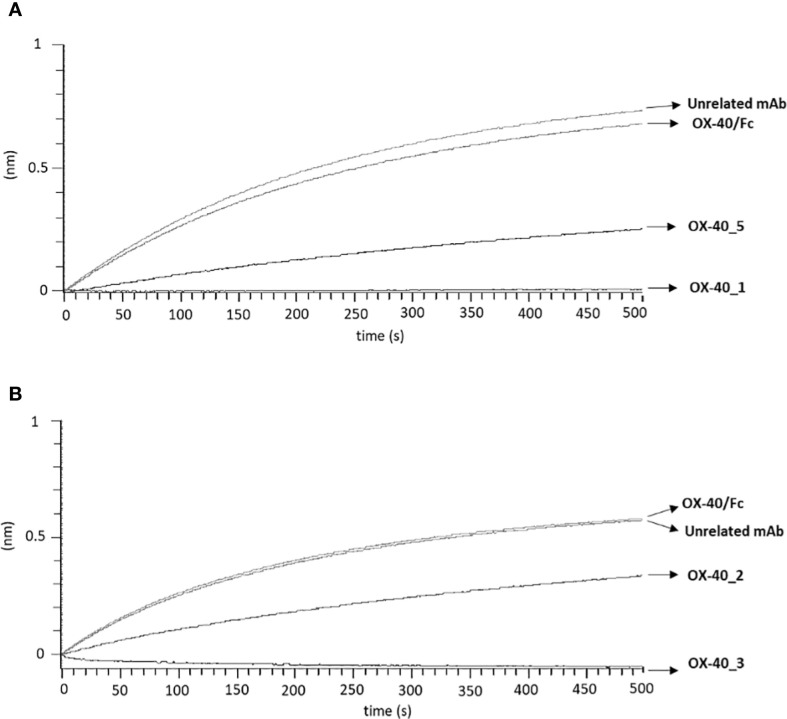
Assessment of competitive binding and interference of novel anti-OX-40 mAbs and OX-40-L. To test the ability of the novel anti-OX-40 mAbs, **(A)** for OX-40_1 and OX-40_5 and **(B)** for OX-40_2 and OX-40_3, to interfere in the OX-40/OX-40L interaction, a BLI analysis was performed. OX40-L/His was used as ligand and immobilized on HIS1K sensor at the concentration of 5 µg/mL and then OX40/Fc receptor was tested at the concentration of 10 nM, before or after preincubation with each indicated anti-OX-40 mAbs (used at 10-fold molar excess). A human unrelated IgG was used as a negative control.

We also decided to investigate whether the epitopes recognized by these new immunoagents overlap or not with that recognized by the ligand OX-40L. To this aim, we performed BLI assays to measure the interference of the new mAbs in the binding between the ligand and the receptor. First, we measured the binding of OX-40 Ligand to its receptor. Briefly, OX40/Fc receptor was immobilized on ProA biosensor at the concentration of 3 µg/mL and OX-40L/His ligand was tested as analyte at increasing concentrations (5–30 nM), the results confirmed its binding affinity for the receptor in the nanomolar range (**Supplementary Figure S4**). Then, to analyze the interference of the mAbs, we immobilized the OX-40L/His ligand at a concentration of 5 μg/ml on HIS1K biosensors; then, we added the recombinant OX-40/Fc receptor at a concentration of 10 nM, before or after an incubation with a 10-fold molar excess of each anti-OX-40 mAb for 1 h 30 min at RT. As a negative control, the OX-40/Fc was also incubated alone or with an unrelated IgG mAb. The results, reported in **Figure 4A** (for OX-40_1 and OX-40_5) and **Figure 4B** (for OX-40_2 and OX-40_3), show that OX-40_1 and OX-40_3 completely impaired the binding between the ligand and the receptor whereas only a partial interference was observed when the receptor was preincubated with OX-40_5 or OX-40_2 mAbs. These findings suggest that OX-40_3 and OX-40_1 are totally blockers and thus they behave as agonists, whereas OX-40_2 and OX-40_5 are only partially agonists or non-agonists.

### 3.5 Bioluminescent cell-based assay to analyze the biological properties of the novel anti-OX-40 mAbs

**Figure 5:**
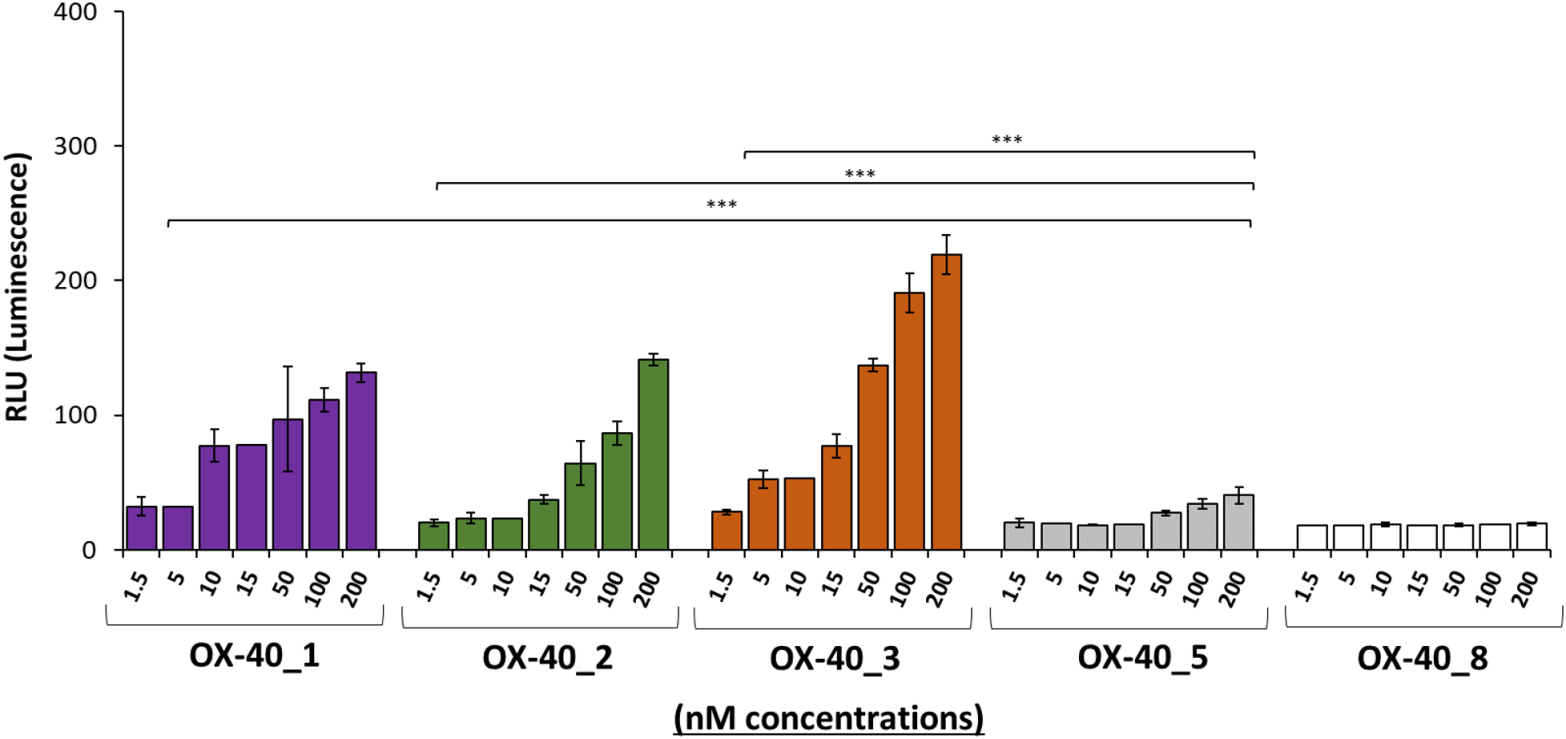
Assessment of the effects of novel anti-OX-40 mAbs on OX-40 intracellular signalling. The ability of the indicated antibodies to activate OX-40 receptor was tested by using OX-40 Bioassay. Genetically engineered Jurkat T cells expressing luciferase reporter gene under the control of OX-40 signalling were used. The cells were treated with the indicated antibodies in a dose escalation range (1.5–200 nM). After treatment, the bioluminescent signal was quantified. OX-40_8 mAb was used as a negative control. Error bars depicted means ± SD and the P values reported is: ***P < 0.001, calculated by Sidak’s multiple comparisons test performed by 2way ANOVA analysis. RLU values at all the doses of the antibody OX-40_1, OX-40_2, and OX-40_3 were compared to those of OX-40_5.

To clarify the different intracellular downstream effects of each mAb, we subjected them to OX-40 reporter cells assay. The assay consists in the stimulation of engineered Jurkat T cells expressing OX-40 receptor associated with a luciferase reporter that generates a bioluminescent signal when stimulated by agonistic molecules. The OX-40 expressing Jurkat T cells were seeded in white 96 well plate, and the following day, were incubated with each of the four antibodies (OX-40_1, 2,_3, 5) at 37 °C at increasing concentrations (1.5–200 nM) for 5 h. The resulting data, represented in **Figure 5**, indicate that OX-40_1, OX-40_2 and OX-40_3 mAbs act as agonists for OX-40 signalling cascade, whereas OX-40_5 does not display an agonistic behaviour. This finding can be explained by its partial interference activity with OX-40 Ligand on the receptor, shown in the **Figure 4**, and could suggest the recognition by this mAb of an epitope not affecting the OX-40 downstream pathway. As a negative control of the assay, the non-specific OX-40_8 mAb was used in parallel and, as expected, it did not show any effect in this system.

**Figure 6:**
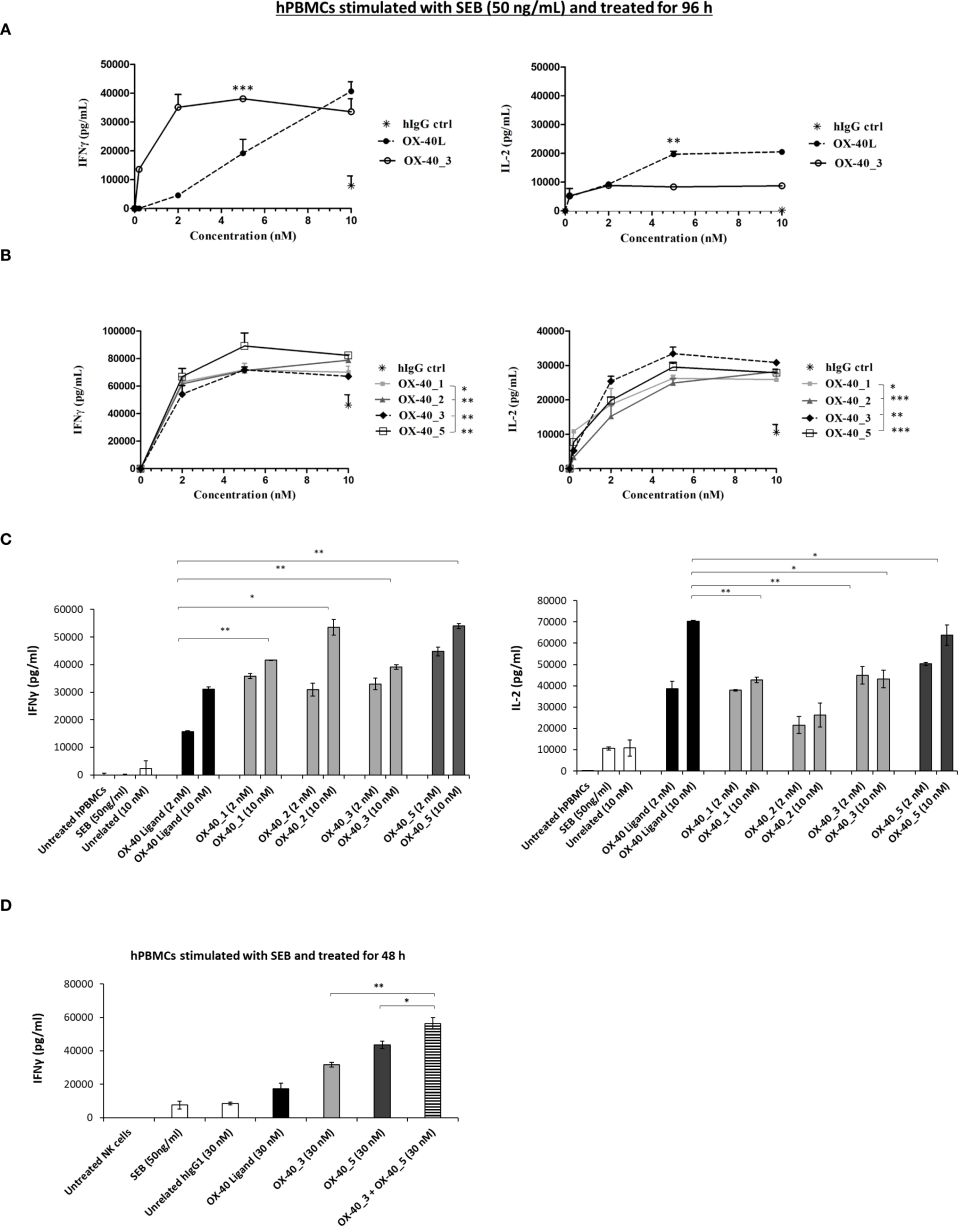
Effects of the novel mAbs on cytokines secretion by hPBMCs. **(A)** hPBMCs were stimulated with SEB (50 ng/mL) and then treated with increasing concentrations (0.2–10 nM) of OX-40_3 (○), or OX-40 Ligand (•) for 96 h The levels of secreted IL-2 and IFNγ were measured by ELISA cytokine secretion test. **(B)** hPBMCs stimulated with SEB (50 ng/mL) were treated with increasing concentrations (0.2–10 nM) of OX-40_1 (▪), OX-40_2 (♦), OX-40_3 (♦), OX-40_5 (□)for 96 h Untreated cells or cells treated with the unrelated human IgG1 (*) were used as negative control. **(C)** IFNγ and IL-2 released by stimulated hPBMCs treated with either OX-40_1, _2, _3 (light grey bars), OX-40_5 (dark grey bars) mAbs or OX-40 Ligand (black bars), used at concentrations of 2 and 10 nM and incubated at 37 °C for 96 h **(D)** SEB-preactivated hPBMCs were treated with OX-40-L (black bar), OX-40_3 (light grey bar),OX-40_5 (dark grey bar), or with OX-40_3/OX-40_5 combination (striped bar) at the concentrations of 30 nM. IFNγ secretion was measured. All the results were obtained by at least three independent experiments. Error bars depicted means ± SD and the P values reported are: ***P < 0.001; **P < 0.01; * ≤ 0.05 by student’s t test (two variables).

Given the stronger agonistic activity shown by OX-40_3 compared with the other antibodies, we decided to compare its effects to the agonistic effect of OX-40 ligand on hPBMCs activation. To this aim, hPBMCs were stimulated with SEB and then treated with either OX-40L or OX-40_3 mAb at increasing concentrations for 96 h. IL-2 and IFNγ cytokines secretion levels were measured in supernatant by ELISA assays. An unrelated IgG1 was used as a negative control. The results, reported in **Figure 6A**, indicate that the ligand strongly increased immune cell proliferation, with a high secretion of IL-2. Indeed, the levels of IL-2, when the cells were treated with the ligand, were doubled in comparison with those observed in the treatment with OX-40_3. On the other hand, OX-40_3 led to a much higher secretion of IFNγ in the supernatant, even at the lowest doses. This difference suggests that the novel agonistic antibodies might exert their biological activity on hPBMCs through a different mechanism compared with that of the ligand, likely mediated also by their Fc region, which is absent in the ligand.

### 3.6 Effects of the novel mAbs on the activation of human peripheral blood lymphocytes

Based on these results, we further extended cytokine secretion profile of all the novel anti OX-40 mAbs in a dose escalation experiment (0.2–10 nM) by using immune cells, as this system mimics the natural environment of human body better than the previous bioluminescence assay. We stimulated hPBMCs with SEB at 50 ng/ml and then treated with each of the anti-OX-40 mAbs. An unrelated IgG1 was used in parallel assays as a negative control. After 96 h, the supernatants were collected and the levels of IFNγ and IL-2 secreted were evaluated by ELISA assays. As shown in **Figure 6B**, the four mAbs were all able to efficiently activate hPBMCs by eliciting secretion of both IL-2 and IFNγ compared to control untreated hPBMCs with an EC_50_ of about 1 nM (**Table 4**). In particular, it is interesting to observe that also OX-40_5 mAb, which did not act as an agonist in the previous bioassay, induced a higher secretion of IFNγ compared to the others, meanwhile OX-40_3, which showed the strongest agonistic activity in the bioluminescent assay, was able to induce the highest levels of IL-2.

**Table 4:** EC_50_ values (nM) of the indicated mAbs in inducing cytokine secretion by hPBMC.

Mab	EC_50_	
	IFNγ	IL-2
OX-40_1	1.18 nM	0.8 nM
OX-40_2	1.25 nM	1 nM
OX-40_3	1.5 nM	1.1 nM
OX-40_5	1.2 nM	1.1 nM

EC_50_ values (nM) of the indicated mAbs in inducing cytokine secretion by hPBMCs. The table reports the EC_50_ values obtained by ELISA cytokines secretion curve analyses with Prism (Graphpad) tool according to the following model: Y = Bmax*X/(Kd+X) + NS*X + Background.

To further investigate on the different mechanisms exploited by the four novel anti-OX-40 mAbs, we decided to compare cytokine secretion profile induced by all the novel mAbs with the one elicited by natural ligand of OX-40 (OX-40-L). Thus hPBMCs, previously stimulated with SEB, were treated with the novel mAbs or with OX-40-L, for 96 h at two different concentrations (2 and 10 nM). As indicated in **Figure 6C**, we observed that the treatments with novel anti-OX-40 mAbs, especially with OX-40_5, were able to induce a higher IFNγ secretion in hPBMCs supernatants than those observed in the treatments with the ligand, likely mediated by a mechanism harnessing the Fc portion of the antibodies (e.g., ADCC or crosslinking via Fc receptors). Conversely, OX-40 ligand induced a higher secretion of IL-2, marker of lymphocytes proliferation. As a further proof for their different mechanisms of action, we investigated the possibility of combining two different non-competitive mAbs, OX-40_3 and OX-40_5, to test their potential additive/synergistic effects at the higher concentration (30nM). As shown in **Figure 6D**, once again, the secretion of IFNγ confirmed to be higher than those of ligand in treatments with novel anti-OX-40 mAbs, especially with OX-40_5, similarly to previous experiments. Interestingly the effects of the combination of these two novel mAbs was stronger than those of single mAbs, thus confirming that they can be combined to potentiate their eventual therapeutic effects.

### 3.7 Effects of anti-OX-40 mAbs on Treg and tumor-cell killing

On the basis of previous observations, we decided to further investigate on the ability of the novel mAbs to induce ADCC, by recruiting the NK cells through the interaction of Fc and CD16 receptor (32). It has been reported in literature that Treg constitutively express high levels of OX-40 and could be targeted by antibodies (33). Thus, we tested the novel mAbs on co-cultures of NK cells with Treg cells (Effector: Target ratio 3:1) or with CD4^+^/CD25^-^ T cells (used as a negative control). All the cell fractions were previously isolated from unfractionated hPBMCs, as described in methods. The two co-cultures were incubated in the absence or presence of the novel anti-OX-40 mAbs at two concentrations (1 and 10 nM) for 48 h and then cell lysis was measured by detecting LDH release in the supernatants.

**Figure 7:**
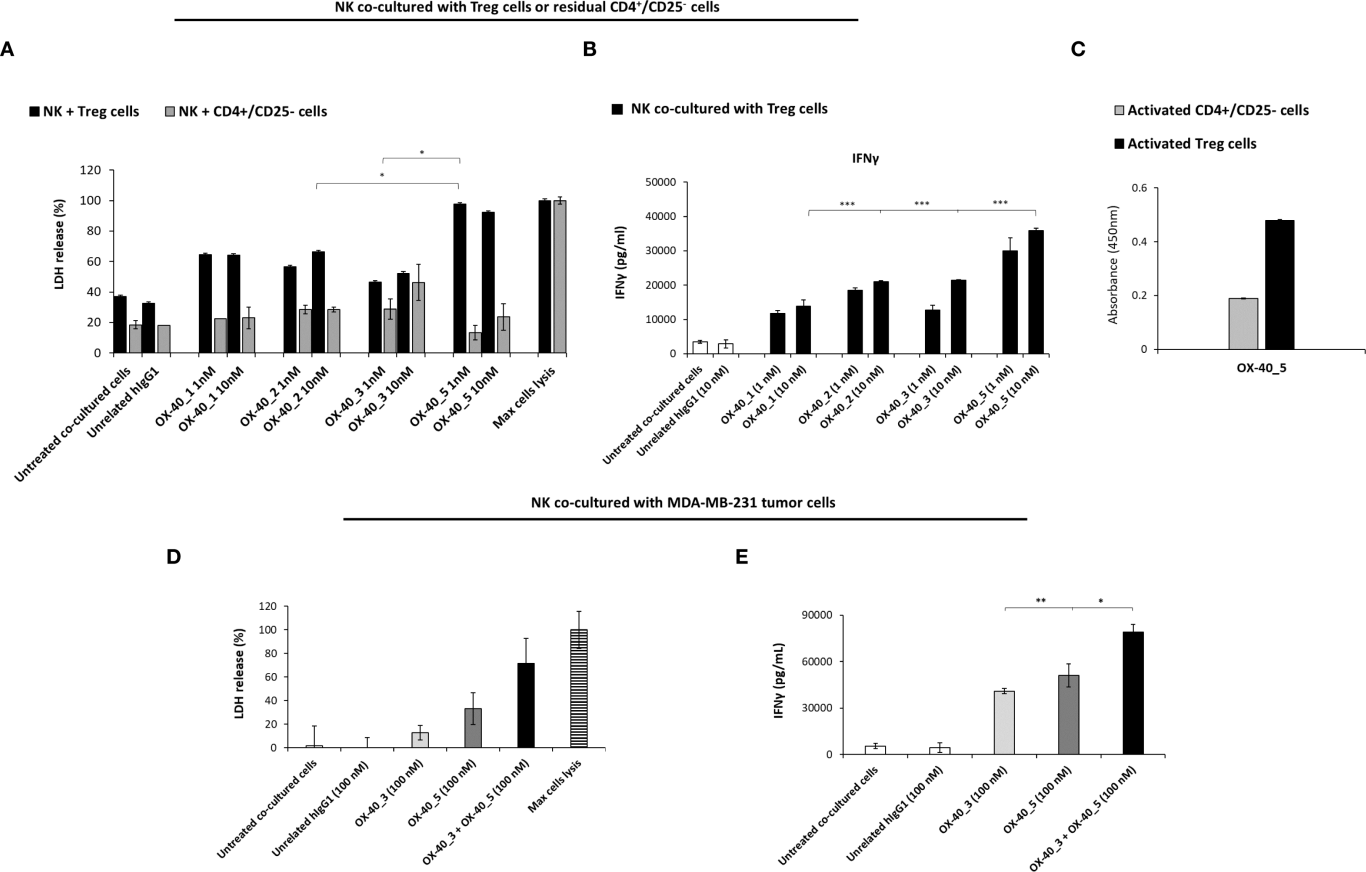
Effects of anti-OX-40 mAbs on co-cultures of NK with Treg, CD4^+^/CD25^-^ cells, or with MDA-MB-231 TNBC cells. **(A)** ADCC induced by anti-OX-40 mAbs at indicated concentration on co-cultures of NK with Treg or with CD4^+^/CD25^-^ T cells, used as a negative control. INFγ secretion was measured by ELISA on supernatant in co-cultures of NK and Treg cells **(B)**, treated as mentioned above. **(C)** Binding of OX-40_5 on Treg and control CD4^+^/CD25^-^ cells was assessed by Cell ELISA. **(D)** ADCC induced by anti-OX-40 mAbs at indicated concentration on co-cultures of NK with MDA-MB-231 tumor cells. **(E)** INFγ secretion was measured by ELISA from supernatant of NK and MDA-MB-231 tumor cells co-cultures. The values were obtained by at least three independent experiments and error bars depicted means ± SD. The P values reported are: ***P < 0.01; **P < 0.01; *P < 0.05, by student’s t test (two variables), calculated by comparing the treatment of the non-agonistic OX-40_5 mAb to each other agonistic mAbs **(A, B)**, or the combination of OX-40_3 with OX-40_5 respect to each one used as single agents **(E)**.

Interestingly, we observed a differential behaviour between agonistic and non-agonistic mAbs: OX-40_5, the non-agonistic mAb, induced a stronger Treg cell lysis, leading to highest levels of LDH released, mediated by NK cells when compared to the agonistic mAbs (**Figure 7A**). This result could explain why OX-40_5 was able to induce hPBMCs activation in previous experiment, even though it lacks agonistic properties. On the other hand, no significant effects were observed when NK cells were co-cultured with control CD4^+^/CD25^-^ cells, confirming their specific ADCC effect on Treg cells. This result was confirmed also by IFNγ level which was found to be higher when the co-culture was treated with OX-40_5 mAb compared to those observed in the other treatments (**Figure 7B**). As a further control, we verified by cell ELISA the differential binding of OX-40_5 in parallel assays on Treg and control CD4^+^/CD25^-^ cells. We found that OX-40_5 recognizes Treg cells with much higher affinity than control cells depleted of Treg, thus confirming that this non-agonistic antibody can preferentially bind to Treg expressing high levels of OX-40 and induce their killing by ADCC (**Figure 7C**).

To test whether the novel anti-OX-40 mAbs induce ADCC mediated by NK cells also on tumor cells, we exploited a co-culture of NK cells with MDA-MB-231 triple negative breast cancer (TNBC) cells. The tumour cell lysis was measured as LDH release in the presence of the OX-40_3 and OX-40_5 (100nM for 72h) or in the presence of unrelated IgG1. Also, combination of the two mAbs was tested. As shown in **Figure 7D**, once again, the non-agonistic OX-40_5 mAb was able to induce highest LDH release and INFγ secretion (**Figure 7E**). More interestingly, its combination with OX-40_3 mAb induced a more potent effect than single agent treatments, confirming that their antitumor effects can be improved when the agonistic and non-agonistic mAbs are used in combination. We also measured the level of IL-2 (data not shown) in the same supernatant of co-cultures of NK and MDA-MB-231 as a further sign of specific activation of NK cells (and not T cells), and, as expected, no significant increases were induced by any of the treatments.

### 3.8 Binding and Effects of novel anti-OX-40 mAbs on isolated NK and CD8^+^ T cells

To further investigate on the different biological behaviour of agonistic and non-agonistic antibodies, we decided to test the novel OX-40 _3 and _5 mAbs on different lymphocytes subpopulations isolated or in co-cultures. To this aim NK, CD8^+^ and CD4^+^ T cells were isolated from unfractionated hPBMCs, by using procedures described in Materials and Methods, and analyzed for their OX-40 expression.

**Figure 8:**
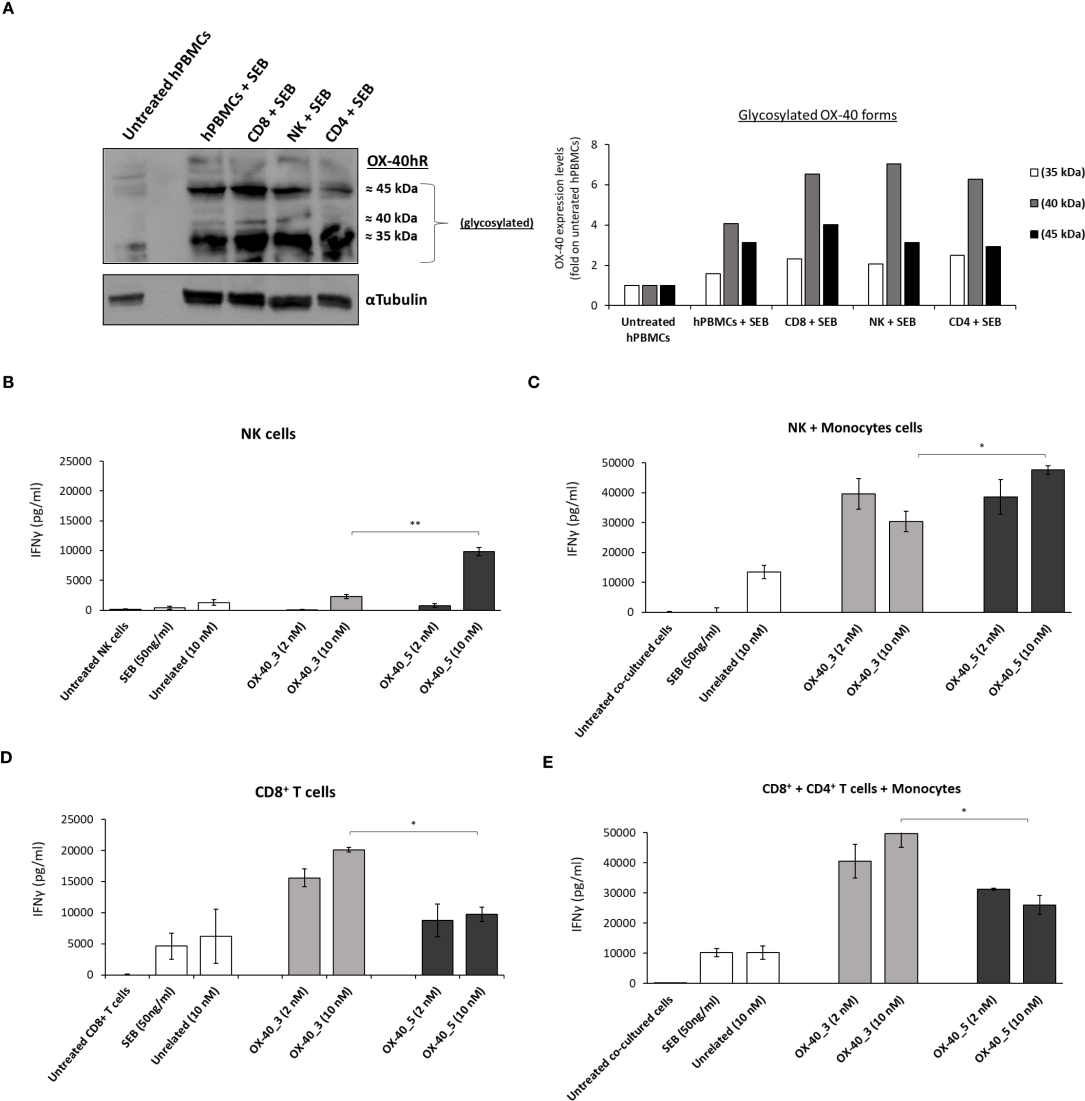
Expression of OX-40 on hPBMCs, NK, CD8^+^ and CD4^+^ T cells and effects of the novel mAbs on these subpopulations. Analyses by WB with a commercial anti-OX-40 antibody of cell extracts from stimulated subpopulations of lymphocytes. The protein levels are expressed as fold increase with respect to untreated cells and the intensity of the bands was normalized to α-tubulin by calculating the ratio of OX-40/tubulin for poorly glycosylated (white bars), medium glycosylated (grey bars) and highly glycosylated (black bars) signal intensities for each cell extract **(A)**. The effects of novel anti-OX-40 mAbs were tested on NK and CD8^+^ cells isolated or in co-cultures with monocytes. IFNγ secretion detected in supernatant of NK cells **(B)** CD8^+^ T cells **(D)** isolated or in co-cultures with monocytes (**C**, **E**) treated with anti-OX-40 mAbs at concentrations of 2 and 10 nM for 48 h at 37°C. Concentration values were obtained by at least three independent experiments and expressed in pg/mL. Error bars depicted means ± SD. The P values reported are: **P < 0.01; *P ≤ 0.05, by student’s t test (two variables), calculated by comparing the non-agonistic OX-40_5 mAb with the agonistic OX-40_3 mAb.

It has been reported in literature that OX-40 is expressed at higher levels on CD4^+^ T cells (34), however as a further check we measured and compared the expression of OX-40 on NK, CD8^+^ and CD4^+^ T cells by analyzing their cell extracts by WB with a commercial anti-OX-40 antibody. We found that the receptor, when expressed in its native conformation on lymphocytes, shows the following glycosylation pattern (**Figure 8A**): a poorly glycosylated form (35 kDa), a medium (40 kDa) and a highly (45 kDa) glycosylated one. We also observed that NK cells express higher levels of the 40 kDa form than CD8^+^ T cells that in turn seem to preferentially express the highly glycosylated (45 kDa) one.

Once confirmed the expression of OX-40 on all the subpopulations, we tested the effects of mAbs on isolated NK and CD8^+^ T cells, or in co-cultures of NK or CD8^+^ T cells with monocytes to analyze the effects on OX-40 stimulation itself or the additional role of Fc engagement, respectively. Briefly, we first activated NK and CD8^+^ T cells with SEB at 50 ng/ml and then treated with OX-40_3 and OX-40_5 at the concentration of 2 and 10 nM for 48 h. An unrelated IgG1 mAb was used in parallel assays as a negative control. As reported in **Figures 8B-E**, OX-40_5 mAb showed the strongest effect on the secretion of INFγ on cultures of NK cells. Similar results were observed in co-cultures of NK plus monocytes where the highest levels of INFγ were released in the presence of OX-40_5 (up to ∼47’600 pg/mL at 10 nM). On the other hand, OX40_3, endowed with an agonistic behaviour, induced a higher IFNγ secretion in CD8+ T cells either isolated or when they were co-cultured with monocytes and CD4+ subpopulations, reaching a concentration of 49’700 pg/mL for the dose of 10 nM. This finding suggests that OX-40_5 seems to better activate NK cells likely due the involvement of Fc portion and CD16 recruitment, compared to OX-40_3 that, instead, showed a more potent CD8^+^ T cell activation.

A cell ELISA with OX-40_3 and OX-40_5 was conducted on unfractionated pre-activated hPBMCs and on isolated subpopulations (CD8^+^ T, CD4^+^ T and NK) to verify whether the two mAbs bind with the same affinity to the target expressed on the different lymphocytes subpopulations and if a differential binding could be responsible for their differential behaviour. As shown in **Supplementary Figure S5**, OX-40_5 showed a slightly higher binding ability for NK cells whereas OX-40_3 for T cells, thus suggesting that the differential behaviour of the two novel agonistic and non-agonistic antibodies could depend both on their binding ability and on their different effects. The agonistic OX-40_3 seems to affect OX-40 signalling on T cells, whereas OX-40_5 devoid of agonistic properties is more prone to activate and recruit NK cells.

To further investigate on the ability of the novel mAbs to recognize the differential glycosylated forms of the native receptor, we analyzed cell extracts of stimulated hPBMCs and NK cells by WB by incubating the membranes with each mAb, OX-40_3, OX-40_5, commercial anti-OX-40 Ab or its natural trimeric ligand fused to Fc, used in parallel for comparison. After detection of the signals, each form was quantified and normalized with α-tubulin. The commercial Ab was used to determine the total expression of OX-40 in hPBMCs and NK cells, (**Supplementary Figures S6A, B**), and shows that the intermediate glycosylated forms (35 and 40 kDa) are the most represented (60% and 80% of total protein, respectively). Accordingly, OX-40_3 and OX-40_5 mAbs showed a similar binding pattern with a higher signal of the same glycosylated forms (about 50%). Surprisingly, these forms were not recognized by the natural trimeric ligand, which preferentially recognized the highly glycosylated form in hPBMCs (≈ 60% of total protein).

These results suggest that the ligand cannot bind to OX-40 on NK cells and seems to recognize the receptor through a sugar epitope present on highly glycosylated form, preferentially expressed by T cells, thus suggesting an important role of glycosylation for OX-40.

To confirm this hypothesis we tested the binding of OX-40 ligand in comparison with that of the novel antibodies on NK cells also by cell ELISA by using the commercial anti-OX-40 Ab to check OX-40 expression level. As shown in **Supplementary Figure S6C**, even though the expression of OX-40 was successfully detected by the commercial Ab, the ligand did not bind to NK cells whereas OX-40_5 bound to these cells more efficiently than OX-40_3.

To further investigate on the role of the highly glycosylated form and its specific recognition by the ligand we analyzed its expression on other non-immune cells, such as normal-like or tumor cells (MDA-MB-231). To this aim, these cells were lysed in parallel with hPBMCs and analyzed by WB with the commercial anti-OX-40 Ab. As shown in **Supplementary Figure S6D**, only the lymphocytes show the protein band corresponding to the highly glycosylated 45 kDa form, which is absent in the extracts of non-immune cells, thus suggesting a critical role of glycosylation for OX-40 and its interaction with the ligand. Finally, we analyzed by WB with the commercial anti-OX40 mAb the glycosylated forms specifically expressed on Treg cells after their isolation from unfractionated hPBMCs. We found that Treg, similarly to the whole lymphocytes show as the main species the protein band corresponding to the highly glycosylated 45 kDa form (**Supplementary Figure S6**), thus confirming that this form is preferentially expressed by all T cell populations.

### 3.9 Comparative analysis of the novel human mAbs and clinically validated Rocatinlimab

Finally, the effects of the novel human anti-OX-40 mAbs were compared with the FDA-approved anti-OX-40 mAb Rocatinlimab. First, we performed epitope binning analyses carried out via BLI. Briefly, recombinant OX-40/Fc receptor was immobilized on a ProA biosensor, then Rocatinlimab was added at a saturating concentration of 200 nM, and each of the novel antibody was added at the same concentration. Rocatinlimab itself was used as a control. The results, reported by the sensorgrams in **Supplementary Figure S7A**, showed that OX-40_2, _3 and _5 are still able to bind to the protein even after the saturation differently from the control antibody used for the saturation, therefore suggesting that the novel antibodies recognize a distinct epitope from that recognized by Rocatinlimab.

We then tested the effects of Rocatinlimab on activation of hPBMCs in comparison with those exerted by OX-40_3 and _5. We stimulated hPBMCs with SEB at 50 ng/ml in the absence or presence of each anti-OX-40 mAb at a concentration of 5 μg/mL. An unrelated IgG1 was used in parallel assays as a negative control. After 48 h, the supernatants were collected and the levels of IFNγ and IL-2 secreted were evaluated by ELISA assays. As shown in **Supplementary Figures S7B, C**, the novel mAbs were able to induce the activation of immune cells by determining a higher release of both IL-2 and IFNγ than those observed with Rocatinlimab. These results suggest that the novel mAbs, recognizing different epitopes from that of Rocatinlimab, are endowed with different biological properties and could represent novel potential therapeutic tools.

### 3.10 Effects of the novel anti−OX-40 mAbs used alone or in combination with anti-PD-L1 mAbs on co-cultures of hPBMCs and tumor cells

In order to evaluate potential synergistic effects with antibodies targeting other inhibitory ICs we investigated the biological effects of the novel anti-OX40 mAbs in combination with the clinically validated anti-PD-L1 mAb, Atezolizumab, in co-cultures of hPBMCs and tumor cells.

**Figure 9:**
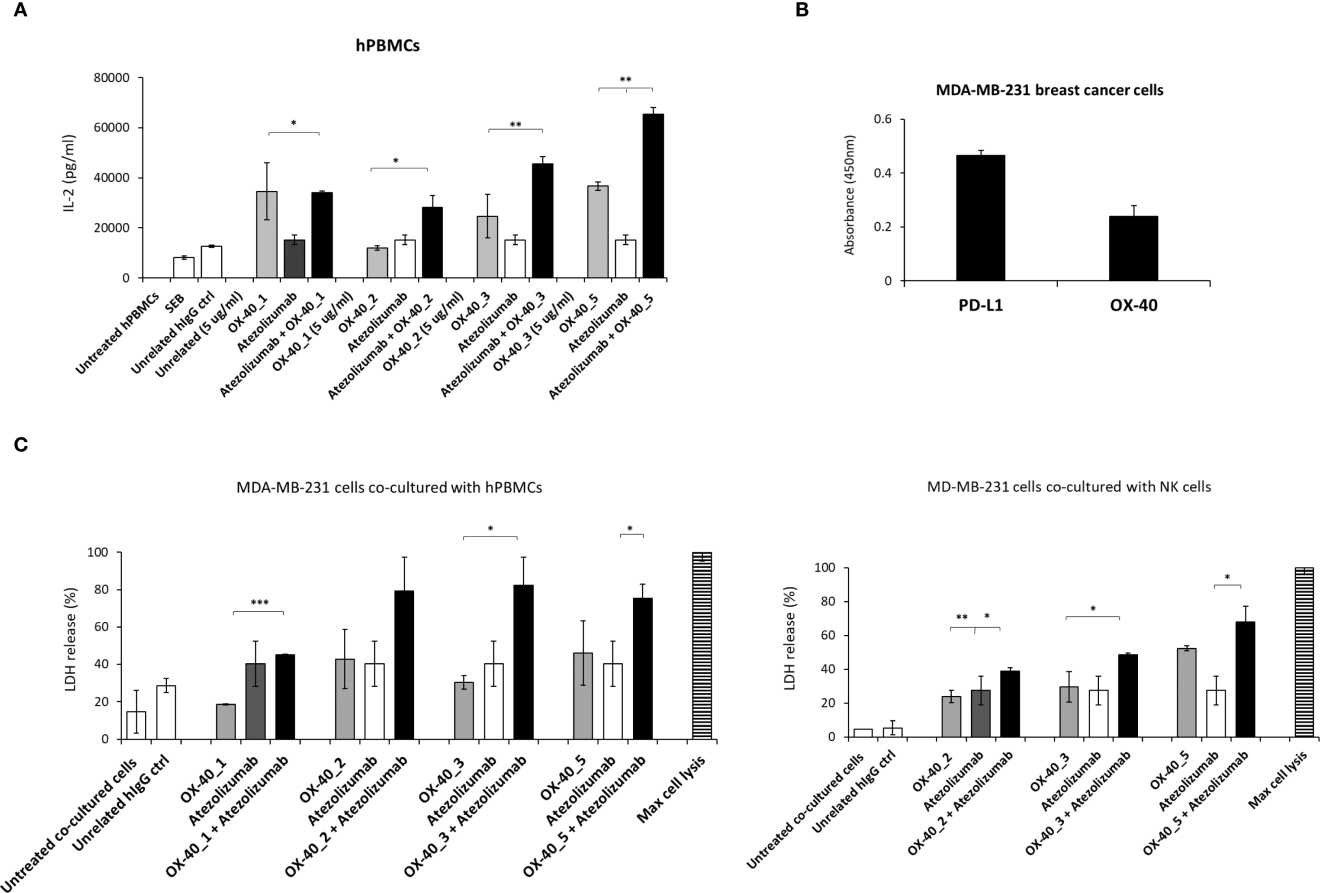
Effects of anti-OX-40 mAbs on co-cultures of lymphocytes and tumor cells used alone or in combination with anti-PD-L1 mAbs. **(A)** IL-2 secretion in supernatants of hPBMCs treated with anti-OX-40 mAbs alone or combined with anti-PD-L1 mAb. IL-2 levels were measured by following the manufacturer’s recommendations of cytokines secretion kit by R & D Systems. **(B)** Analysis of OX-40 and PD-L1 expression on different tumor cell lines by cell ELISA. **(C)** Tumor cell lysis induced by OX-40 mAbs alone or in combination with anti-PD-L1 mAbs on co-cultures of MDA-MB-231 tumor cells with unfractionated hPBMCs (left) or NK cells (right). The results were obtained by at least three independent experiments. Error bars depicted means ± SD and The P values reported are: ***P < 0.001; **P < 0.01; * ≤ 0.05, by student’s t test (two variables), calculated by comparing the combinations with each respective compound used as a single agent.

First, we compared the effects of the novel anti-OX-40 mAbs when used alone or in combination with Atezolizumab on the proliferation of human lymphocytes. To this aim, hPBMCs were stimulated with SEB (50 ng/mL) and treated with anti-OX-40 mAbs alone or in combination with Atezolizumab for 96 h at the concentration of 5 μg/mL. As shown in **Figure 9A**, 3 out of four novel mAbs (OX-40_2, _3, _5) combined with Atezolizumab showed higher levels of secreted IL-2. In particular, the treatments of Atezolizumab with OX-40_3 and OX-40_5, showed the highest ability to induce proliferation of hPBMCs with an IL-2 secretion up to 40’000 and 60’000 pg/mL, respectively. These results suggest that it is possible to combine OX-40 and PD-L1 targeting mAbs to obtain a more potent effect.

On the basis of these results, we decided to further study the effects of these combinations on co-cultures of tumor cells and hPBMCs or NK cells, in order to evaluate their anti-tumor effects. Once checked the expression levels of OX-40 and PD-L1 on MDA-MB-231 breast tumor cell line by cell ELISA assays, by using a commercial anti-OX-40 Ab (**Figure 9B**), wetested the novel mAbs in co-cultures with human lymphocytes or NK cells. To this aim, tumor cells were plated at the density of 10’000/well and incubated with hPBMCs or NK cells (effector: target ratio 5:1) preactivated with SEB (50 ng/mL) for 48 h and then treated with anti-OX-40 mAbs alone or in combination with the validated anti-PD-L1 mAb for 48 h. The supernatants were collected and then cell lysis was evaluated by measuring LDH release in the supernatant. As shown in **Figure 9C**, the combination of Atezolizumab with OX-40_2, OX-40_3 or OX-40_5 exhibits stronger effects, in comparison with single mAbs treatments also in co-cultures with tumor cells, in line with the previous results shown on non-fractionated lymphocytes.

## 4 Discussion

Immune checkpoint inhibitors, such as monoclonal antibodies targeting the PD-1/PD-L1 axis and CTLA-4, have significantly improved the efficacy of cancer therapy, and the development of this type of tools has emerged as one of most active field of research (1–3). Addressing the challenges posed by innate or acquired resistance to these therapies often require combinations of mAbs to overcome resistance to monotherapy, which often occurs in the tumor microenvironment (2). Among these, agonistic antibodies enhancing the activity of co-stimulatory receptors have emerged as potential tools for inducing immune responses and counteracting immune suppression. OX-40 (CD134), a member of the tumor necrosis factor receptor superfamily (TNFRSF), is mainly found on activated T cells (4). The stimulation of OX-40 pathway via its ligand OX-40L or agonistic antibodies promotes T cell growth, longevity, and memory development (5–8). The anti-OX-40 mAbs showed favorable safety and tolerability profile in First-In-Human Phase I studies but following clinical trials have generally yielded suboptimal results, particularly in later-stage studies (35, 36). One contributing factor is the intricate biology of the OX-40 pathway and its varied effects on different immune cells. This has led to the engineering of antibody isotypes tailored to act by specific functions and targeting particular pathways. The presence of OX-40 on Treg cells, for example, has driven the use of antibody isotypes that trigger antibody-dependent cellular cytotoxicity (36, 37). Isotype selection is also influenced by the need for Fc receptor-mediated cross-linking, which supports the formation of immune complexes crucial for effective OX-40 signalling (37). Furthermore, the interplay between therapeutic antibodies and the body’s natural OX-40 ligand adds another layer of complexity, which is still under investigation. These challenges highlight the ongoing need for the development of novel OX-40 agonists with unique and optimized biological profiles.

We previously adopted a phage display selection approach on both OX-40-expressing cells in its native conformation and recombinant protein to isolate novel antibody fragments targeting OX-40 (24–26). The integration of phage display with a high-throughput next-generation sequencing-based screening strategy enabled the rapid identification of four distinct antibody sequences capable of binding to OX-40 with high affinity. These human antibody fragments were subsequently converted into full-length IgG1 antibodies.

In this study, we fully characterized these four antibodies, named OX-40_1, OX-40_2, OX-40_3, and OX-40_5, by evaluating their binding to OX-40, by using both ELISA and BLI, and testing their ability to interfere in the interaction between the OX-40 receptor and its ligand. Among them, OX-40_1 and OX-40_3 behaved as full agonists, effectively competing with the natural ligand for OX-40 binding. In contrast, OX-40_2 and OX-40_5 exhibited only partial competition, reflecting differences in epitope recognition. It is particularly interesting to note that assays analyzing various aspects of OX-40 pathway activation provide the opportunity to diversify the impact of different antibodies. Indeed, despite all identified antibodies binding at sub-nanomolar concentrations, their biological effects are highly diverse. This is especially evident when comparing the effects of antibodies with the natural ligand in their ability to induce IL-2 and IFNγ secretion. Specifically, regarding IL-2 secretion, the ligand exhibits a stronger effect than the novel mAbs whereas it shows an opposite effect for IFNγ secretion, as the treatment with the novel antibodies, especially OX-40_5, leads to the strongest secretion. Furthermore, competition with the natural ligand, and consequent agonistic behaviour of the antibodies, was not found to be a discriminating factor in hPBMC activation.

As previously mentioned, OX-40_5, differently from the other antibodies, did not behave as an OX-40 agonist, and appeared nearly inert in the functional bioluminescence assay, which is based on OX-40 specific downstream signalling. On the other side, it was the most potent in inducing IFNγ release following the stimulation of NK cells in cytotoxic activity against Treg cells or TNBC cells in ADCC assays. Therefore, its anti-tumor potential might derive from its ability to trigger an efficient Treg cells depletion thanks to the high levels of OX-40 on Treg cells (33, 38, 39), its ability to bind with high affinity to Treg and the recruitment of the activating FcγRs on NK cells by its Fc (32), similarly to the mechanism of action observed in a previous study involving a different anti-OX-40 monoclonal antibody (40). Thus, we can conclude that agonistic OX-40_2 and _3 induce T cell activation by mimicking the ligand, stimulating OX-40 signalling and increasing more efficiently IL-2 release, whereas non-agonistic OX-40_5 induces more efficiently the activation of NK cells and consequent release of IFNγ also by this cell population (see **Supplementary Figure S8**). Furthermore, in Western Blotting analyses of immune cell extracts, OX-40_5 recognizes all different forms of OX-40 receptor, including the one more abundant in NK cell extracts, whereas the ligand preferentially recognizes the higher molecular weight band corresponding to the highly glycosylated form of the receptor. It is known from literature and Protein Data Bank (PDB) (41, 42) that OX-40 contains glycans based on 2-acetoamido-2-deoxy-beta-D-glucopyranose, linked to two asparagine residues (N146 and N160). Here we found, for the first time, that this highly glycosylated form, expressed by hPBMCs and T reg, was present at much lower levels in NK cells where, instead, marked expression levels of a moderately glycosylated form with a lower molecular weight was observed, unlike the other T cell subpopulations. Interestingly, this form is not recognized by the ligand which is not able to affect NK cells differently from the novel mAbs.

Furthermore, considering the different mechanisms of action of OX-40–3 and OX-40_5, the combination of agonistic and non-agonistic mAbs resulted in a more potent activation of hPBMCs, especially when NK cells were co-cultured with TNBC cells. This combination could also promote the generation of memory T cells due to the effect of agonistic anti-OX-40 mAb (15, 43) and, simultaneously, the long-term NK cell activation mediated by non-agonistic anti-OX-40, for a long-lasting protection against tumor relapse. Moreover, epitope binning analyses show that they recognize distinct epitopes not overlapping with that of the only FDA-approved Rocatinlimab to treat atopic dermatitis, thus providing new tools to target OX-40 with different biological properties (11).

Finally, combining these antibodies with a clinical inhibitor of the PD-1/PD-L1 immune axis, Atezolizumab, resulted in an additive capacity to induce inflammatory cytokine secretion and tumor cell killing in co-culture assays.

These findings provide valuable insights into the therapeutic potential of anti-OX-40 mAbs in modulating immune responses against tumors. By targeting OX-40 with agonistic antibodies, this study demonstrates the ability to potentiate anti-tumor immunity while reducing the suppressive function of Tregs, thus shifting the immune landscape toward a more pro-inflammatory and tumor-eliminating state. In summary, this study extends beyond the description of novel monoclonal antibodies. First, it demonstrates the applicability of an NGS-based screening method that leverages activated human cells and recombinant protein bait to isolate and enrich antibody species recognizing the antigen in its native form. Additionally, it highlights the complexity of the OX-40 pathway, where competition with the natural ligand and the ability to induce ADCC are key factors to be considered in future studies. Furthermore, it confirms that the combinations of antibodies recognizing different epitopes of OX-40 or targeting OX-40 and PD-L1 lead to stronger anti-tumor effects. Finally, it suggests for the first time a critical role of extensive glycosylation of OX-40 in T cells, not observed in NK or other non-immune cells, for the specific recognition by the ligand.

## Data Availability

The original contributions presented in the study are included in the article/**Supplementary Material**. Further inquiries can be directed to the corresponding author.
