# Supplementary material for: Generation of novel human anti-OX-40 mAbs endowed with different biological properties as tools for cancer therapy
Source: Front Immunol. 2025 Sep 17;16:1644391. doi: 10.3389/fimmu.2025.1644391 (PMC12484235; doi:10.3389/fimmu.2025.1644391)
Supplement: Supplementary file 1 [file DataSheet1.pdf]

Supplementary Material

1 Supplementary Figures

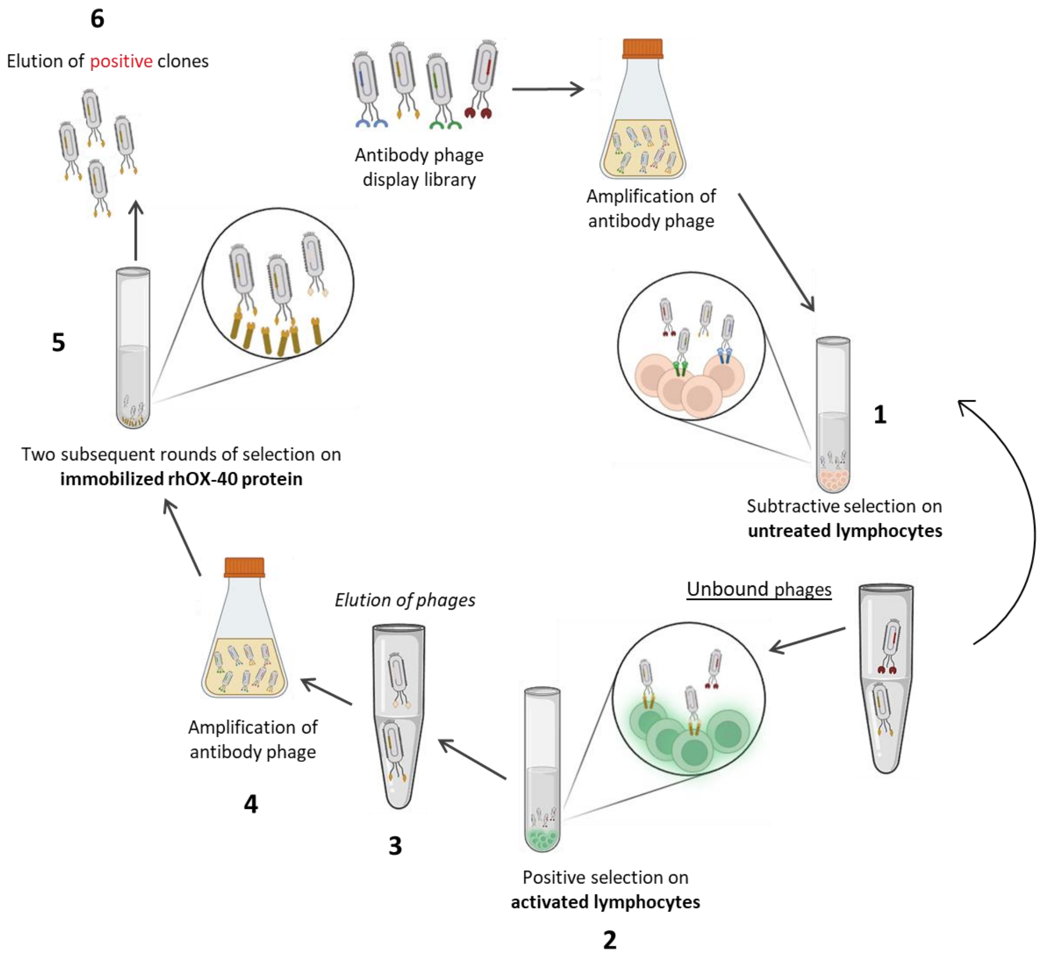

Supplementary Figure 1. Graphical representation of phage display selection of anti-OX-40 positive clones. Each round of selection included 4 following steps: panning, washes, elution and amplification. Phages were recovered from the antibody phage display library, amplified and used for a subtractive selection on untreated hPBMCs (**1**), followed by a positive selection on activated hPBMCs (**2**). The eluted phages (**3**) were amplified (**4**) and subjected to two subsequent rounds of selection on immobilized hrOX-40/Fc coated protein (**5**). The phages were then eluted (**6**) and the screening of positive clones performed by NGS.

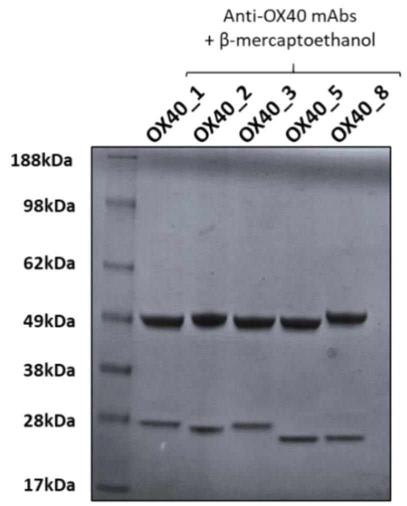

Supplementary Figure 2. Analysis of purity by SDS-PAGE and Coomassie Staining of the novel purified anti-OX-40 mAbs. The novel monoclonal antibodies were generated by transfecting HEK293EBNA cells and purified by Pro-A affinity chromatography from conditioned medium, as described in methods. The analysis of purity and stability of the mAbs was performed by SDS-PAGE under reducing conditions, by adding β-mercaptoethanol.

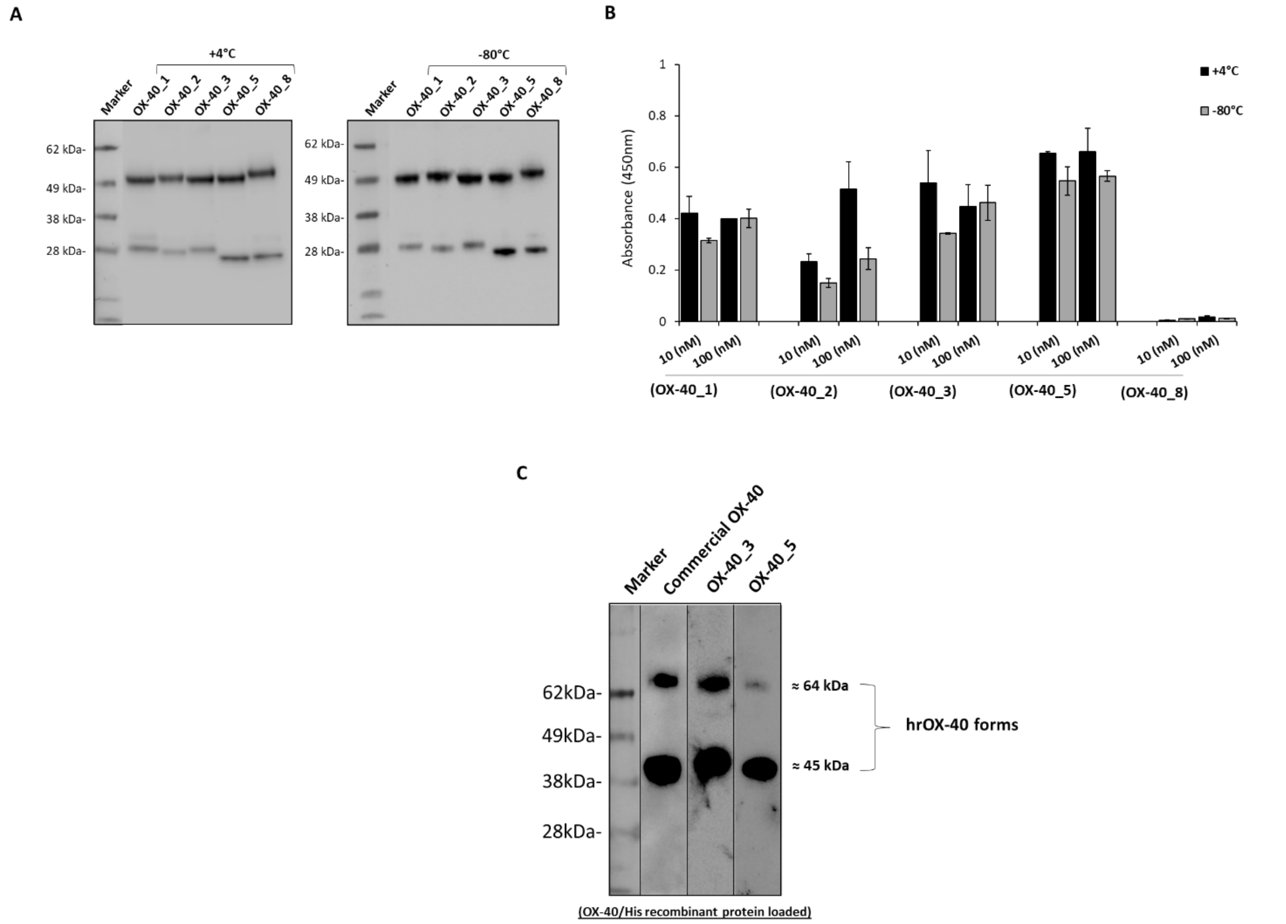

Supplementary Figure 3. Analysis of stability of anti-OX-40 mAbs up to 1 month of storage in different conditions. (**A**) Western Blotting analysis under reducing conditions of the novel anti-OX-40 mAbs tested after one month at 4°C or after one cycle of freezing and thawing out at -80°C. The signal was detected by using a mixture of HRP-conjugated anti-Fab and anti-Fc mAbs. (**B**) Binding by ELISA of novel purified mAbs tested at two concentrations (10, 100 nM) on immobilized OX-40/Fc recombinant protein after one month of storage at 4°C (black bars) or after one cycle of freezing and thawing out at -80°C (grey bars). (**C**) The binding specificity was tested on Human OX-40/His recombinant protein analysed by WB, by incubating the membrane in parallel with the commercial anti-OX-40 Ab or the novel mAbs. The signal was detected by using the HRP-conjugated anti-rabbit or anti-Fc, respectively.

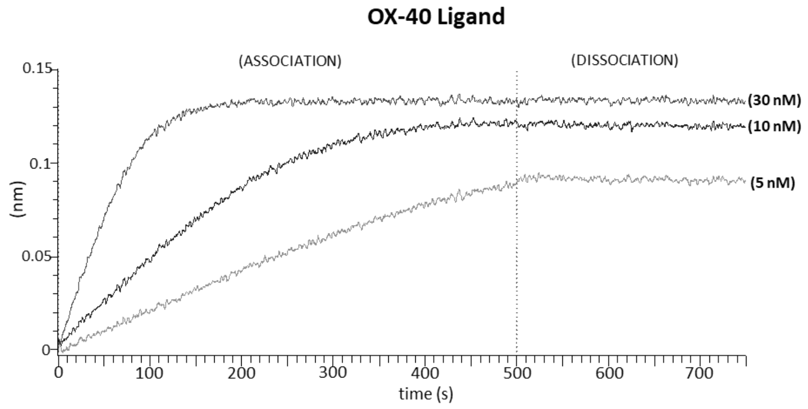

Supplementary Figure 4. Binding of OX-40 Ligand on immobilized OX-40 receptor via BLI analyses. The sensorgram reports association and dissociation rates of the human recombinant OX-40L/His ligand tested as analyte (10-30 nM) on human recombinant OX-40/Fc ligand immobilized on ProA sensor (3 µg/mL).

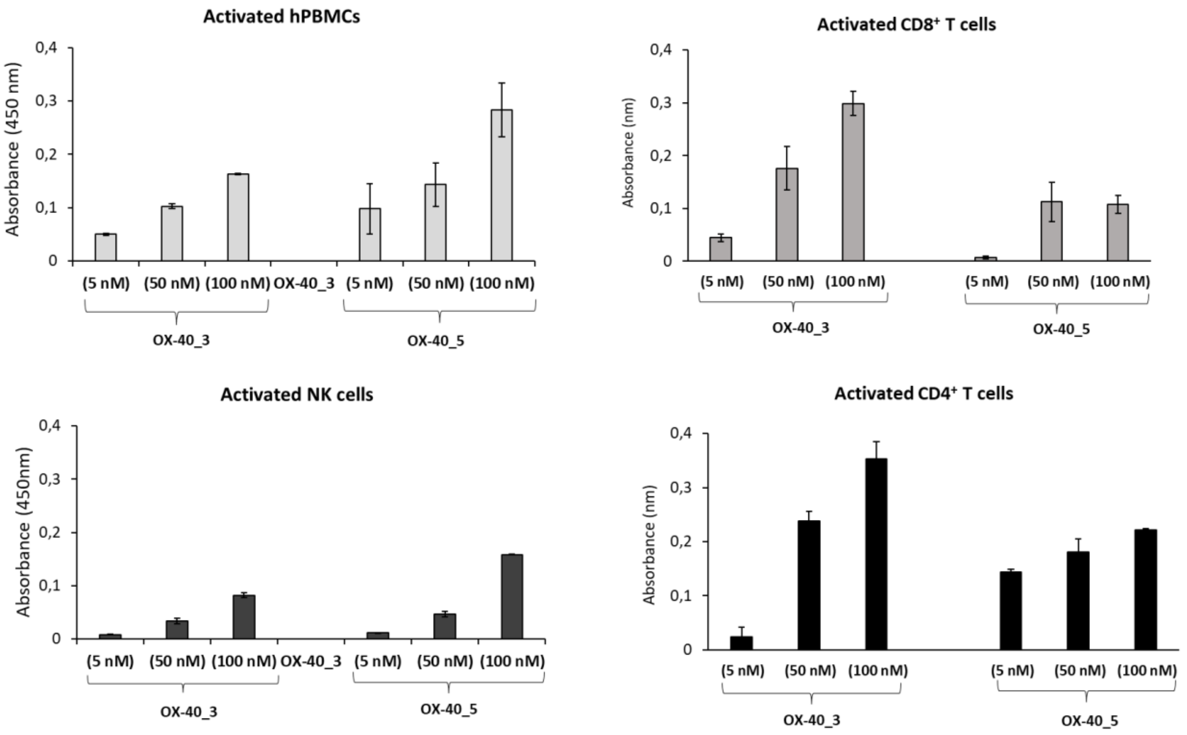

Supplementary Figure 5. Binding of the novel human anti-OX-40 antibodies on different immune cell populations. The binding ability of OX-40_3 and _5 at increasing concentrations (5-100 nM) was determined on activated hPBMCs, NK, CD8+ and CD4+ T cell subpopulations. The signal was detected by incubation with a secondary anti-fab HRP-conjugated. Absorbance values were reported as the mean of at least three determinations and error bars depicted means ± SD.

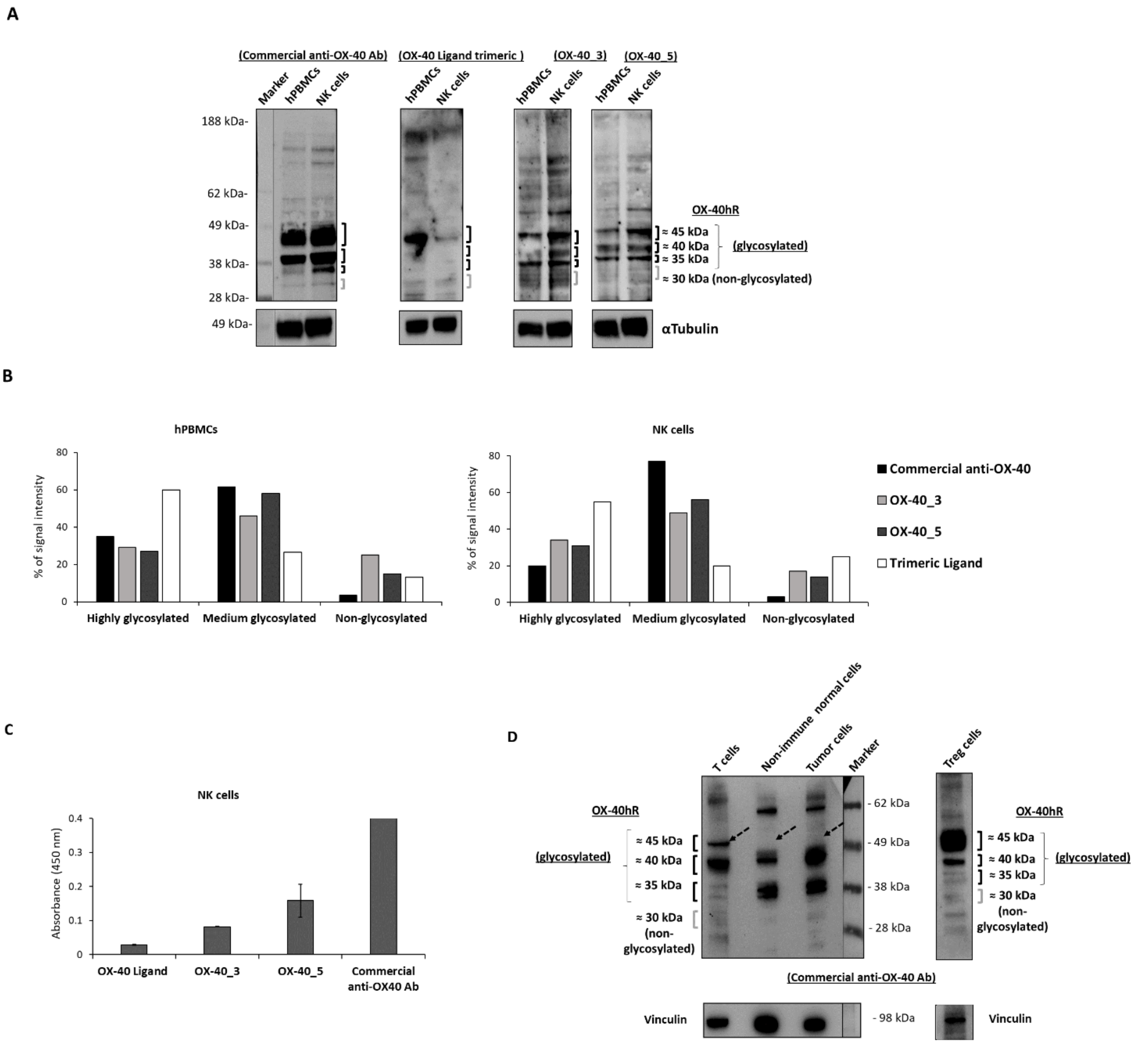

Supplementary Figure 6. Binding of the novel mAbs to the differentially glycosylated forms of OX-40. (**A**) hPBMCs or NK cells ($2 \cdot 10^6$) were stimulated with SEB (50 ng/ml) for 48 h and then NK cells were isolated as described in Methods. The cell extracts were analysed by WB and the membrane incubated in parallel with OX-40_3, OX-40_5, the commercial Ab or trimeric Ligand/Fc, for comparison. The signals were detected by using their corresponding HRP-conjugated secondary Abs, and the protein levels were normalized on α-tubulin. (**B**) The histograms report the levels of each non-glycosylated or glycosylated forms expressed as % of the total OX-40 signal. (**C**) Cell ELISA assays were performed to evaluate, in parallel, the binding ability of OX-40_3, OX-40_5 and OX-40L/Fc on NK cells. The binding was detected with an anti-Fc HRP-conjugated secondary antibody. Absorbance values were reported as the mean of at least three determinations and error bars depicted means ± SD. **(D)** Cell lysates from hPBMCs, Treg, non-immune normal or tumor cells were analyzed via WB by incubating the membrane with the commercial anti-OX-40 Ab, to evaluate the levels of different OX-40 forms levels in each cell type. The dashed arrows indicate the highly glycosylated 45 kDa form.

The signal was detected by using an anti-rabbit HRP-conjugated secondary antibody, and vinculin was used for normalization.

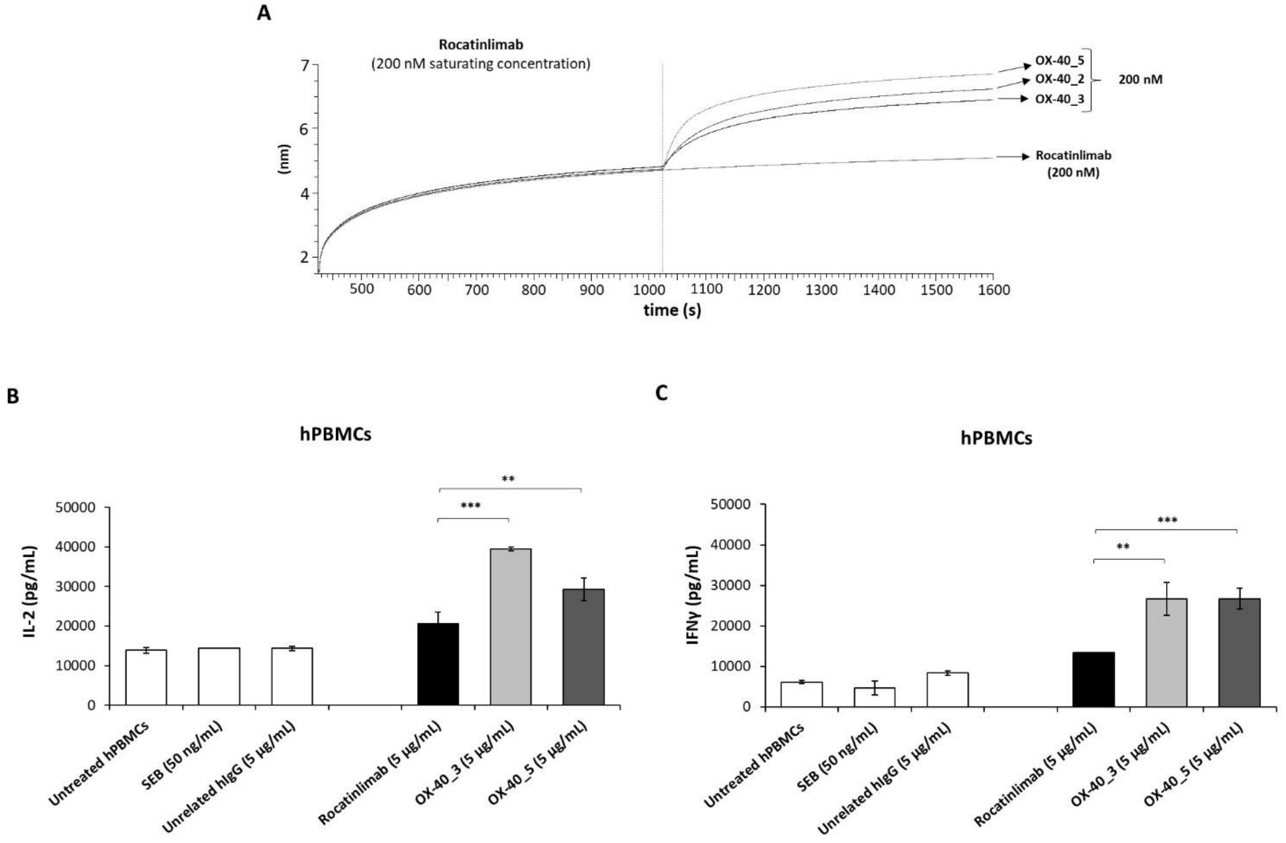

Supplementary Figure 7. Comparison between the novel anti-OX-40 mAbs and the clinically validated Rocatinlimab. (**A**) Tandem BLI assays were performed by immobilizing OX40/Fc protein on ProA biosensor at a concentration of 3 µg/mL, then the protein was saturated with Rocatinlimab (200 nM). After that, the subsequent binding of OX-40_2, OX-40_3 or OX-40_5 mAbs at the concentration of 200 nM was measured. In parallel, Rocatinlimab was tested again as a negative control to verify the biosensor saturation. (**B-C**) Activation of stimulated hPBMCs treated with Rocatinlimab (black), OX-40_3 (light grey bars) or OX-40_5 (dark grey bars). Cells untreated or treated with the unrelated human IgG1 were used as negative controls (white bars). The levels of IFNγ and IL-2 induced by the treatments were evaluated by ELISA assays. Error bars depicted ± SD and the P values reported are *** $P < 0.001$; ** $P < 0.01$, obtained by using student's t test (two variables) and by comparing the novel OX-40_3 and OX-40_5 with the clinically validated Rocatinlimab.

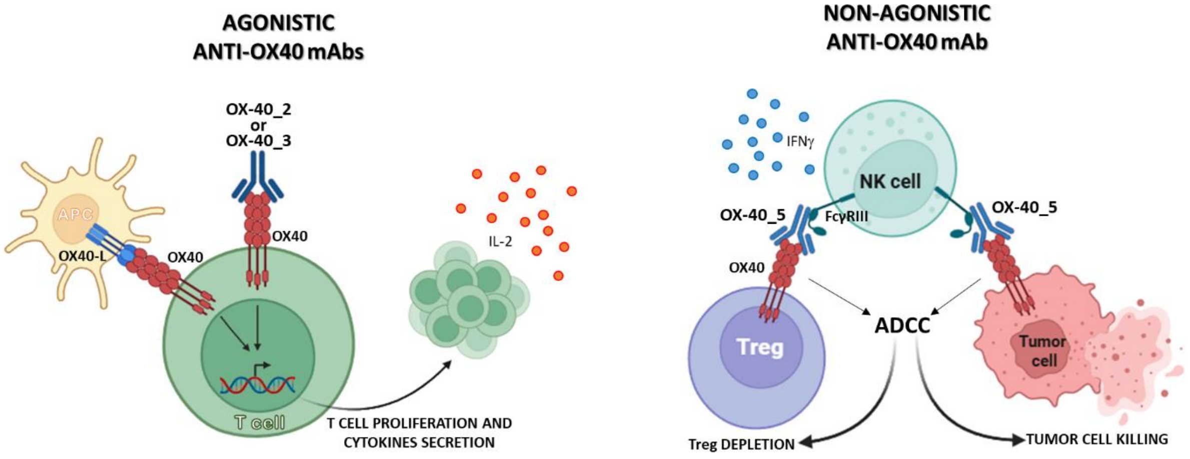

Supplementary Figure 8. Model of mechanisms of action of the novel anti-OX-40 mAbs. Schematic representation of the biological effects exerted by agonistic antibodies (left panel) and non-agonistic antibodies (right panel).
